# Clinical prediction models for mortality in patients with covid-19: external validation and individual participant data meta-analysis

**DOI:** 10.1136/bmj-2021-069881

**Published:** 2022-07-12

**Authors:** Valentijn M T de Jong, Rebecca Z Rousset, Neftalí Eduardo Antonio-Villa, Arnoldus G Buenen, Ben Van Calster, Omar Yaxmehen Bello-Chavolla, Nigel J Brunskill, Vasa Curcin, Johanna A A Damen, Carlos A Fermín-Martínez, Luisa Fernández-Chirino, Davide Ferrari, Robert C Free, Rishi K Gupta, Pranabashis Haldar, Pontus Hedberg, Steven Kwasi Korang, Steef Kurstjens, Ron Kusters, Rupert W Major, Lauren Maxwell, Rajeshwari Nair, Pontus Naucler, Tri-Long Nguyen, Mahdad Noursadeghi, Rossana Rosa, Felipe Soares, Toshihiko Takada, Florien S van Royen, Maarten van Smeden, Laure Wynants, Martin Modrák, the CovidRetro collaboration, Folkert W Asselbergs, Marijke Linschoten, CAPACITY-COVID consortium, Karel G M Moons, Thomas P A Debray

**Affiliations:** 1 Julius Center for Health Sciences and Primary Care, University Medical Centre Utrecht, Utrecht University, Utrecht, Netherlands; 2 Cochrane Netherlands, University Medical Centre Utrecht, Utrecht University, Netherlands; 3 Data Analytics and Methods Task Force, European Medicines Agency, Amsterdam, Netherlands; 4 Dirección de Investigación, Instituto Nacional de Geriatría, Mexico City, Mexico; 5 MD/PhD (PECEM) Program, Faculty of Medicine, National Autonomous University of Mexico, Mexico City, Mexico; 6 Maxima MC, Veldhoven, the Netherlands; 7 Bernhoven, Uden, Netherlands; 8 Department of Development and Regeneration, KU Leuven, Leuven, Belgium; 9 Department of Biomedical Data Sciences, Leiden University Medical Centre, Leiden, Netherlands; 10 EPI-centre, KU Leuven, Leuven, Belgium; 11 Department of Cardiovascular Sciences, College of Life Sciences, University of Leicester, Leicester, UK; 12 John Walls Renal Unit, University Hospitals of Leicester NHS Trust, Leicester, UK; 13 School of Population Health and Environmental Sciences, King’s College London, London, UK; 14 Faculty of Chemistry, Universidad Nacional Autónoma de México, México City, Mexico; 15 Centre for Clinical Infection and Diagnostics Research, School of Immunology and Microbial Sciences, King’s College London, London, UK; 16 Department of Respiratory Sciences, College of Life Sciences, University of Leicester, Leicester, UK; 17 NIHR Leicester Biomedical Research Centre, University of Leicester, Leicester, UK; 18 Institute for Global Health, University College London, London, UK; 19 Department of Respiratory Medicine, University Hospitals of Leicester NHS Trust, Leicester, UK; 20 Department of Infectious Diseases, Karolinska University Hospital, Stockholm, Sweden; 21 Division of Infectious Diseases, Department of Medicine Solna, Karolinska Institute, Stockholm, Sweden; 22 Copenhagen Trial Unit, Centre for Clinical Intervention Research, Department 7812, Rigshospitalet, Copenhagen University Hospital, Denmark; 23 Laboratory of Clinical Chemistry and Haematology, Jeroen Bosch Hospital, Den Bosch, Netherlands; 24 Department of Health Technology and Services Research, Technical Medical Centre, University of Twente, Enschede, Netherlands; 25 Department of Cardiovascular Sciences, College of Life Sciences, University of Leicester, Leicester, UK; 26 Heidelberger Institut für Global Health, Universitätsklinikum Heidelberg, Germany; 27 University of Iowa Carver College of Medicine, Iowa City, IA, USA; 28 Centre for Access and Delivery Research Evaluation Iowa City Veterans Affairs Health Care System, Iowa City, IA, USA; 29 Section of Epidemiology, Department of Public Health, University of Copenhagen, Copenhagen, Denmark; 30 Department of Pharmacy, University Hospital Centre of Nîmes, Nîmes, France; 31 Division of Infection and Immunity, University College London, London, UK; 32 Infectious Diseases Service, UnityPoint Health-Des Moines, Des Moines, IA, USA; 33 Industrial Engineering Department, Universidade Federal do Rio Grande do Sul, Porto Alegre, Brazil; 34 Department of General Medicine, Shirakawa Satellite for Teaching And Research (STAR), Fukushima Medical University, Fukushima, Japan; 35 Department of Epidemiology, CAPHRI Care and Public Health Research Institute, Maastricht University, Maastricht, Netherlands; 36 Institute of Microbiology of the Czech Academy of Sciences, Prague, Czech Republic; 37 Department of Cardiology, Division of Heart and Lungs, University Medical Centre Utrecht, Utrecht University, Utrecht, Netherlands; 38 Health Data Research UK and Institute of Health Informatics, University College London, London, UK; 39 Institute of Cardiovascular Science, Faculty of Population Health Sciences, University College London, London, UK

## Abstract

**Objective:**

To externally validate various prognostic models and scoring rules for predicting short term mortality in patients admitted to hospital for covid-19.

**Design:**

Two stage individual participant data meta-analysis.

**Setting:**

Secondary and tertiary care.

**Participants:**

46 914 patients across 18 countries, admitted to a hospital with polymerase chain reaction confirmed covid-19 from November 2019 to April 2021.

**Data sources:**

Multiple (clustered) cohorts in Brazil, Belgium, China, Czech Republic, Egypt, France, Iran, Israel, Italy, Mexico, Netherlands, Portugal, Russia, Saudi Arabia, Spain, Sweden, United Kingdom, and United States previously identified by a living systematic review of covid-19 prediction models published in *The BMJ*, and through PROSPERO, reference checking, and expert knowledge.

**Model selection and eligibility criteria:**

Prognostic models identified by the living systematic review and through contacting experts. A priori models were excluded that had a high risk of bias in the participant domain of PROBAST (prediction model study risk of bias assessment tool) or for which the applicability was deemed poor.

**Methods:**

Eight prognostic models with diverse predictors were identified and validated. A two stage individual participant data meta-analysis was performed of the estimated model concordance (C) statistic, calibration slope, calibration-in-the-large, and observed to expected ratio (O:E) across the included clusters.

**Main outcome measures:**

30 day mortality or in-hospital mortality.

**Results:**

Datasets included 27 clusters from 18 different countries and contained data on 46 914patients. The pooled estimates ranged from 0.67 to 0.80 (C statistic), 0.22 to 1.22 (calibration slope), and 0.18 to 2.59 (O:E ratio) and were prone to substantial between study heterogeneity. The 4C Mortality Score by Knight et al (pooled C statistic 0.80, 95% confidence interval 0.75 to 0.84, 95% prediction interval 0.72 to 0.86) and clinical model by Wang et al (0.77, 0.73 to 0.80, 0.63 to 0.87) had the highest discriminative ability. On average, 29% fewer deaths were observed than predicted by the 4C Mortality Score (pooled O:E 0.71, 95% confidence interval 0.45 to 1.11, 95% prediction interval 0.21 to 2.39), 35% fewer than predicted by the Wang clinical model (0.65, 0.52 to 0.82, 0.23 to 1.89), and 4% fewer than predicted by Xie et al’s model (0.96, 0.59 to 1.55, 0.21 to 4.28).

**Conclusion:**

The prognostic value of the included models varied greatly between the data sources. Although the Knight 4C Mortality Score and Wang clinical model appeared most promising, recalibration (intercept and slope updates) is needed before implementation in routine care.

## Introduction

Covid-19 has had a major impact on global health and continues to disrupt healthcare systems and social life. Millions of deaths have been reported worldwide since the start of the pandemic in 2019.^1^ Although vaccines are now widely deployed, the incidence of SARS-CoV-2 infection and the burden of covid-19 remain extremely high. Many countries do not have adequate resources to effectively implement vaccination strategies. Also, the timing and sequence of vaccination schedules are still debatable, and virus mutations could yet hamper the future effectiveness of vaccines.^2^

Covid-19 is a clinically heterogeneous disease of varying severity and prognosis.^3^ Risk stratification tools have been developed to target prevention and management or treatment strategies, or both, for people at highest risk of a poor outcome.^4^ Risk stratification can be improved by the estimation of the absolute risk of unfavourable outcomes in individual patients. This involves the implementation of prediction models that combine information from multiple variables (predictors). Predicting the risk of mortality with covid-19 could help to identify those patients who require the most urgent help or those who would benefit most from treatment. This would facilitate the efficient use of limited medical resources, and reduce the impact on the healthcare system—especially intensive care units. Furthermore, if a patient’s risk of a poor outcome is known at hospital admission, predicting the risk of mortality could help with planning the use of scarce resources. In a living systematic review (update 3, 12 January 2021; www.covprecise.org), 39 prognostic models for predicting short term (mostly in-hospital) mortality in patients with a diagnosis of covid-19 have been identified.^5^

Despite many ongoing efforts to develop covid-19 related prediction models, evidence on their performance when validated in external cohorts or countries is largely unknown. Prediction models often perform worse than anticipated and are prone to poor calibration when applied to new individuals.^6^ ^7^ ^8^ Clinical implementation of poorly performing models leads to incorrect predictions and could lead to unnecessary interventions, or to the withholding of important interventions. Both result in potential harm to patients and inappropriate use of medical resources. Therefore, prediction models should always be externally validated before clinical implementation.^9^ These validation studies are performed to quantify the performance of a prediction model across different settings and populations and can thus be used to identify the potential usefulness and effectiveness of these models for medical decision making.^7^ ^8^ ^10^ ^11^ ^12^ We performed a large scale international individual participant data meta-analysis to externally validate the most promising prognostic models for predicting short term mortality in patients admitted to hospital with covid-19.

## Methods

### Review to identify covid-19 related prediction models

We used the second update (21 July 2020) of an existing living systematic review of prediction models for covid-19 to identify multivariable prognostic models and scoring rules for assessing short term (at 30 days or in-hospital) mortality in patients admitted to hospital with covid-19.^5^ During the third update of the living review (12 January 2021),^13^ additional models were found that also met the study eligibility criteria of this individual participant data meta-analysis, which we also included for external validation.

**Fig 1:**
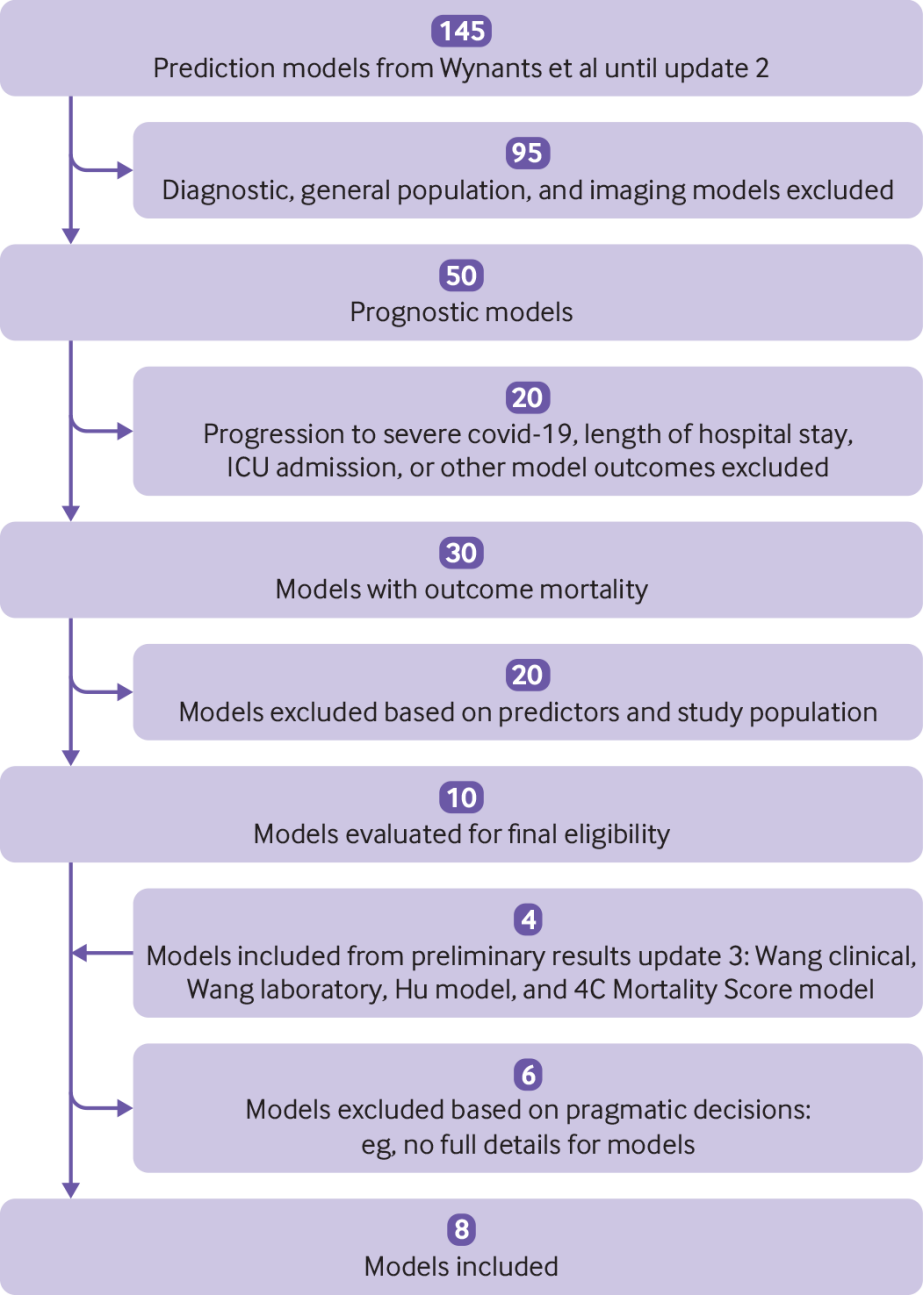
Flowchart of inclusion of prognostic models. The second update took place on 21 July 2020. ICU=intensive care unit

We considered prediction models to be eligible for the current meta-analysis if they were developed using data from patients who were admitted to a hospital with laboratory confirmed SARS-CoV-2 infection. In papers that reported multiple prognostic models, we considered each model for eligibility. As all the prognostic models for covid-19 mortality in the second update (21 July 2020) of the living systematic review had a lower quality and high risk of bias in at least one domain of PROBAST (prediction model study risk of bias assessment tool),^7^ ^8^ we only excluded models that had a high risk of bias for the participant domain and models for which applicability was deemed poor, as well as imaging based algorithms (see fig 1).

### Review to identify patient level data for model validation

We searched for individual studies and registries containing data from routine clinical care (electronic healthcare records), and data sharing platforms with individual patient data of those admitted to hospital with covid-19. We further identified eligible data sources through the second update (21 July 2020) of the living systematic review.^5^ ^13^ In addition, we consulted the PROSPERO database, references of published prediction models for covid-19, and experts in prognosis research and infectious diseases.

Data sources were eligible for model validation if they contained data on mortality endpoints for consecutive patients admitted to hospital with covid-19. We included only patients with a polymerase chain reaction confirmed SARS-CoV-2 infection. We excluded patients with no laboratory data recorded in the first 24 hours of admission. In each data source, we adopted the same eligibility criteria for all models that we selected for validation. We used 30 days for the scoring rule by Bello-Chavolla et al^14^ when available, otherwise in-hospital mortality was used (see table 1).

### Statistical analyses

For external validation and meta-analysis we used a two stage process.^15^ ^16^ The first stage consisted of imputing missing data and estimating performance metrics in individual clusters. For datasets that included only one hospital (or cohort) we defined the cluster level as the individual hospital (or cohort). In the CAPACITY-COVID dataset,^17^ which contains data from multiple countries, we considered each country as a cluster. For the data from UnityPoint Hospitals in Iowa, United States, we considered each hospital as a cluster. We use the term cluster throughout the paper. In the second stage we performed a meta-analysis of the performance metrics.^18^ ^19^ We did not perform an a priori sample size calculation, as we included all data that we found through the review and that met the inclusion criteria.

#### Stage 1: Validation

We imputed sporadically missing data 50 times by applying multiple imputation (see supplementary material B). Using each of the eight models, we calculated the mortality risk or mortality score of all participants, in clusters where the respective models’ predictors were measured in at least some of the participants. Subsequently, we calculated the concordance (C) statistic, observed to expected ratio (O:E ratio), calibration slope, and calibration-in-the-large for each model in each imputed cluster.^11^ The C statistic is an estimator for the probability of correctly identifying the patient with the outcome in a pair of randomly selected patients of which one has developed the outcome and one has not.^20^ The O:E ratio is the ratio of the number of observed outcomes divided by the number of outcomes expected by the prediction model. The calibration slope is an estimator of the correction factor the prediction model coefficients need to be multiplied with, to obtain coefficients that are well calibrated to the validation sample.^11^ ^21^ The calibration-in-the-large is an estimator for the (additive) correction to the prediction model’s intercept, while keeping the prediction model’s coefficients fixed.^11^ ^21^ Supplementary material B provides details of the model equations.

#### Stage 2: Pooling performance

In the second stage of the meta-analysis, we pooled the cluster specific logit C statistic, calibration slope, and log O:E ratios from stage 1.^22^ We used restricted maximum likelihood estimation and the Hartung-Knapp-Sidik-Jonkman method to derive all confidence intervals.^23^ ^24^ To quantify the presence of between study heterogeneity, we constructed approximate 95% prediction intervals, which indicated probable ranges of performance expected in new clusters.^25^ We performed the analysis in R (version 4.0.0 or later, using packages mice, pROC, and metamisc) and we repeated the main analyses in STATA.^26^ ^27^ ^28^ ^29^ ^30^ This study is reported following the Transparent Reporting of a multivariable prediction model for Individual Prognosis Or Diagnosis (TRIPOD) checklist for prediction model validation (see supplementary material C).^31^ ^32^

#### Sensitivity analysis

None of the datasets contained all predictors, meaning the models could not all be validated in a single dataset, which hampered the interpretation. As such, for each performance measure taken separately, we performed a meta-regression on all performance estimates where we included country (not cluster, to save degrees of freedom) and model as predictors (both as dummy variables), which we had not prespecified in our protocol. Then we used these meta-models to predict the performance (and 95% confidence intervals) of each prediction model in each included country, thereby allowing for a fairer comparison of the performance between models. All R code is available from github.com/VMTdeJong/COVID-19_Prognosis_IPDMA.

### Patient and public involvement

Patients and members of the public were not directly involved in this research owing to lack of funding, staff, and infrastructure to facilitate their involvement. Several authors were directly involved in the treatment of patients with covid-19, have been in contact with hospital patients with covid-19, or have had covid-19.

## Results

### Review of covid-19 related prediction models

We identified six prognostic models and two scoring rules that met the inclusion criteria (fig 1). Table 1 summarises the details of the models and scores. The score developed by Bello-Chavolla et al predicted 30 day mortality,^14^ whereas the other score and the six models predicted in-hospital mortality.^33^ ^34^ ^35^ ^36^ ^37^

**Table 1:** Overview of selected models for predicting short term mortality in patients admitted to hospital with SARS-CoV-2 infection

Model	Country of development	Development population	Predicted outcome	Predictors	Model type	Estimation method
Bello-Chavolla et al^14^	Mexico	All reported confirmed cases of covid-19, including hospital admission, ICU admission, and outpatient treatment.	30 day mortality	Age, diabetes (type 2), obesity (clinician- defined), pneumonia, chronic kidney disease, chronic obstructive pulmonary disease, immunosuppression	Score	Rounding of Cox regression coefficients (unpenalised)
Xie et al^33^	China	Adults (≥18 years) with confirmed covid-19, admitted in officially designated covid-19 treatment centres	In-hospital mortality	Age, lactate dehydrogenase, lymphocyte count, oxygen saturation	Prediction model	Logistic regression (unpenalised)
Hu et al^34^	China	Patients with severe covid-19 in Tongji Hospital, which specifically accommodated for people with covid-19. Patients directly admitted to intensive care unit were excluded. Patients with certain comorbidities (including cancer, uraemia, aplastic anaemia) were also excluded. Patients with a short hospital stay (<7 days) were excluded	In-hospital mortality	Age, high sensitivity C reactive protein, D-dimer, lymphocyte count	Prediction model	Logistic regression (unpenalised)
Zhang et al DCS and DCSL models^35^	China	Adults (≥18 years) admitted to two hospitals	In-hospital mortality	DCS model: Age, sex, diabetes (unspecified), immunocompromised, malignancy, hypertension, heart disease, chronic kidney disease, cough, dyspnoea DCSL model: Age, sex, chronic lung disease, diabetes (unspecified), malignancy, cough, dyspnoea, neutrophil count, lymphocyte count, platelet count, C reactive protein, creatinine	Prediction model	Logistic regression (lasso penalty)
Knight et al 4C Mortality Score^36^	UK	Adults (≥18 years) admitted across 260 hospitals	In-hospital mortality	Age, sex, number of comorbidities (chronic cardiac disease, respiratory disease, renal disease, liver disease, neurological conditions; dementia; connective tissue disease; diabetes (type 1 and 2); AIDS/HIV; malignancy, obesity), respiratory rate, oxygen saturation (room air), Glasgow coma scale score, urea, C reactive protein	Score	Rounding of logistic regression coefficients (lasso penalty)
Wang et al clinical and laboratory models^37^	China	Adults (≥18 years) admitted to hospital. Pregnant women were excluded	In-hospital mortality	Clinical model: Age, history of hypertension, history of heart disease Laboratory model: Age, oxygen saturation, neutrophil count, lymphocyte count, high sensitivity C reactive protein, D-dimer, aspartate aminotransferase, glomerular filtration rate	Prediction model	Logistic regression (unpenalised). Intercept from nomogram

The six prognostic models were estimated by logistic regression. The Bello-Chavolla score and Knight et al 4C Mortality Score were (simplified) scoring rules that could be used to stratify patients into risk groups. The Bello-Chavolla score was developed with Cox regression, whereas the 4C Mortality Score was developed with lasso logistic regression and its weights were rescaled and rounded to integer values.

Although the 4C Mortality Score itself does not provide absolute risks, these were available through an online calculator. As the authors promoted the use of the online calculator, we have used these risks in our analysis. For two models by Wang et al (clinical and laboratory), no intercept was available and were approximated.^38^

### Review of patient level data for model validation

**Fig 2:**
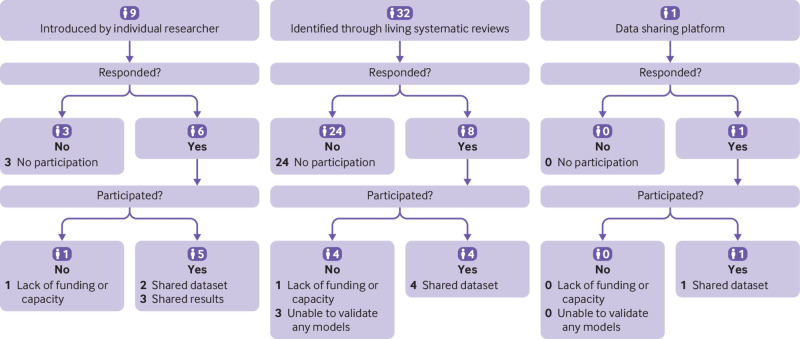
Flowchart of data sources

We identified 10 data sources, including four through living systematic reviews, one through a data sharing platform, and five by experts in the specialty (fig 2). The obtained datasets included 27 clusters from 18 different countries and contained data on 46 701 patients, 16 418 of whom died (table 2). Study recruitment was between November 2019 and April 2021. Most clusters included all patients with polymerase chain reaction confirmed covid-19, although in some clusters only patients admitted through a specific department were included (see supplementary material A, table S1). Mean age ranged from 45 to 71 years.

**Table 2:** Characteristics of the included external validation cohorts and clusters

Dataset, cluster	No of patients	Start recruitment date	End recruitment date	Total No (%) of deaths	Mean (SD) age; (IQR) (years)	No (%) male
Karolinska Institute, Sweden	1670	27 Feb 2020	1 Sep 2020	193 (11.56)	57.30 (18.70); (43-71)	983 (58.90)
Albert Einstein Hospital, Brazil	453	27 Feb 2020	25 Jun 2020	17 (3.75)	56.44 (14.92); (46-68.50)	295 (65.05)
Czech Republic Academy of Sciences, Czech Republic	213	3 Mar 2020	12 Oct 2020	42 (20)*	68.56 (16.56); (58-80)	105 (49)
University College London, UK	411	1 Feb 2020	30 Apr 2020	115 (28)	66 (53-79)†	252 (61.31)
General Directorate of Epidemiology, Mexico:						
All data, from this source	28 176	1 Mar 2020	16 Apr 2020	12 990 (46.10)	58.57 (15.93); (48-70)	17 019 (60.40)
Development cohort excluded	25 056			11 556 (46.12)	59.08 (15.94); (49-70)	15 035 (60.01)
Tongji Hospital,^39^ China	332	10 Jan 2020	18 Feb 2020	155 (46.69)	58.98 (16.65); (46-70)	198 (59.64)
CAPACITY-COVID:						
Belgium	221	12 Feb 2020	14 Oct 2020	51 (23.08)*	68.14 (15.72); (57-81)	137 (61.99)
Egypt	45	12 Apr 2020	12 Aug 2020	9 (20)	60.89 (14.37); (50-73)	21 (46.67)
France	46	13 Feb 2020	18 Dec 2020	3 (6.52)*	67.96 (12.52); (62-77)	34 (73.91)
Iran	90	10 Feb 2020	5 May 2020	13 (14.44)*	63.19 (15.39); (53.25-73)	59 (65.56)
Israel	25	10 Apr 2020	9 Aug 2020	2 (8)	50.36 (20.15); (31-67)	14 (56)
Italy	106	6 Feb 2020	4 May 2020	22 (20.75)	70.72 (11.63); (62.25-78.75)	72 (67.92)
Netherlands	5100	22 Nov 2019	30 Jul 2020	1003 (19.67)*	66.37 (14.15); (57-76)	3172 (62.20)
Portugal	44	25 Mar 2020	19 Aug 2020	10 (22.73)*	71.43 (13.32); (63.75-82)	30 (61.18)
Russia	278	22 Apr 2020	4 Jun 2020	19 (6.83)	60.09 (15.49); (50.25-71)	137 (49.28)
Saudi Arabia	389	29 Feb 2020	24 Sep 2020	57 (14.65)*	50.56 (16.79); (38-62)	270 (69.41)
Spain	47	5 Mar 2020	20 Apr 2020	10 (21.28)*	70.98 (16.68); (55-83.75)	28 (59.57)
Jeroen Bosch Ziekenhuis, Netherlands	383	9 Mar 2020	29 Dec 2020	154 (40.21)	70.21 (14.79); (61-81)	226 (59.01)
Iowa (USA), UnityPoint Hospitals:						
Hospital 1	288	3 Mar 2020	31 Jul 2020	14 (4.86)	46.49 (19.29); (32-59)	147 (51.04)
Hospital 2	929	3 Mar 2020	31 Jul 2020	67 (7.21)	50.12 (22.54); (33-68)	454 (48.87)
Hospital 3	95	3 Mar 2020	31 Jul 2020	6 (6.32)	45.31 (20.54); (27-59)	45 (47.37)
Hospital 4	66	3 Mar 2020	31 Jul 2020	6 (9.09)	48.77 (21.26); (31-65)	39 (59.09)
Hospital 5	511	3 Mar 2020	31 Jul 2020	22 (4.31)	51.03 (20.63); (35-67)	240 (46.97)
Hospital 6	393	3 Mar 2020	31 Jul 2020	22 (5.60)	45.35 (19.02); 30-60	176 (44.78)
Hospital 7	295	3 Mar 2020	31 Jul 2020	18 (6.10)	47.60 (20.10); 32-63	162 (54.92)
Leicester covTrack, UK	3908	Jan 2020	Apr 2021	110 (28.22)	63.29 (19.19); (50-79)	2063 (52.79)
King’s College Hospital, UK	2400	28 Feb 2020	28 Mar 2021	295 (12.29)	59.76 (20.58); (47-76)	1314 (54.75)

SD=standard deviation; IQR=interquartile range.

* Observed number of deaths before multiple imputation.

† Median (IQR).

### External validation and meta-analysis

All results are presented in supplementary material D. The Wang clinical model could be validated in 24 clusters (see supplementary table S2), followed by Hu et al’s model, which was validated in 16 clusters (see supplementary table S3). The remaining models were less often validated, as predictor measurements were available in fewer datasets: the Bello-Chavolla score was validated in seven clusters (see supplementary table S4), the model by Xie et al in nine clusters (see supplementary table S5), the DCS model by Zhang et al in six clusters (see supplementary table S6), the DCSL model by Zhang et al in six clusters (see supplementary table S7), the 4C Mortality Score in six clusters (see supplementary table S8), and the Wang laboratory model in three clusters (see supplementary table S9).

### Discrimination

**Fig 3:**
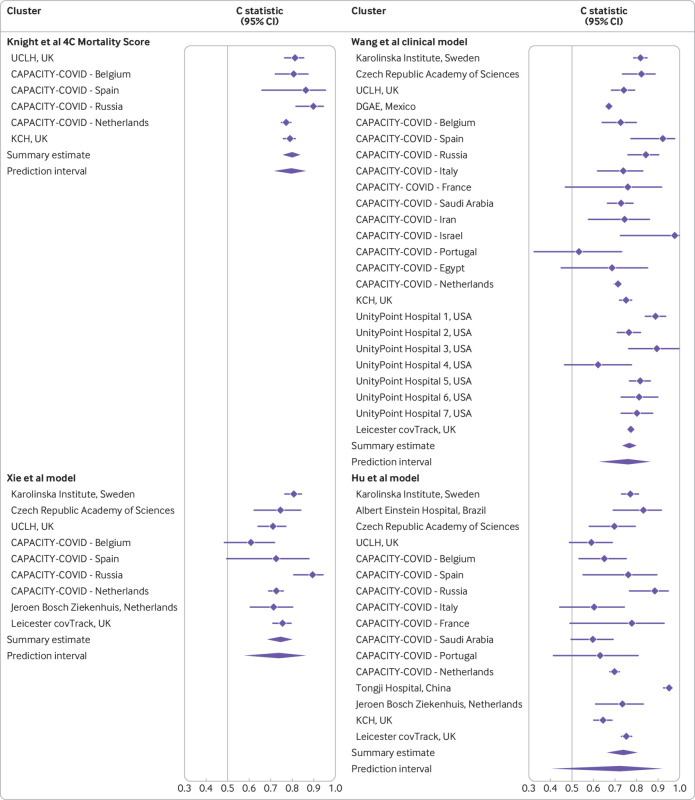
Pooled C statistic estimates with corresponding 95% confidence interval and approximate 95% prediction intervals for four models (see supplementary file for full data). The Knight et al 4C Mortality Score had a C statistic of 0.786 (95% confidence interval 0.78 to 0.79) in the development data and 0.767 (0.76 to 0.77) in the validation data in the original publication. The Wang et al clinical model had a C statistic of 0.88 (0.80 to 0.95) in the development data and 0.83 (0.68 to 0.93) in the validation data in the original publication. The Xie et al model had a C statistic of 0.89 (0.86 to 0.93) in the development data, 0.88 after optimism correction, and 0.98 (0.96 to 1.00) in the validation data in the original publication. The Hu et al model had a C statistic of 0.90 in the development data and 0.88 in the validation data in the original publication. UCLH=University College London; DGAE=General Directorate of Epidemiology; KCH=King’s College Hospital

The 4C Mortality Score showed the highest discrimination, with a pooled C statistic of 0.80 (95% confidence interval 0.75 to 0.84, fig 3 and see supplementary fig S4). The heterogeneity of discrimination of this model across datasets (95% prediction interval 0.72 to 0.86) was low compared with that of the other models. The next best discriminating model was the Wang clinical model, with a pooled C statistic of 0.77 (0.73 to 0.80, fig 3), and a greater heterogeneity (95% prediction interval 0.63 to 0.87). Two other models attained a summary C statistic >0.70: the Xie model with a C statistic of 0.75 (0.68 to 0.80, 95% prediction interval 0.58 to 0.86, fig 3), and the Hu model with a C statistic of 0.74 (0.66 to 0.80, 95% prediction interval 0.41 to 0.92, fig 3). The summary C statistic estimates for the remaining models were <0.70 (see supplementary fig S1).

### Calibration: observed to expected

**Fig 4:**
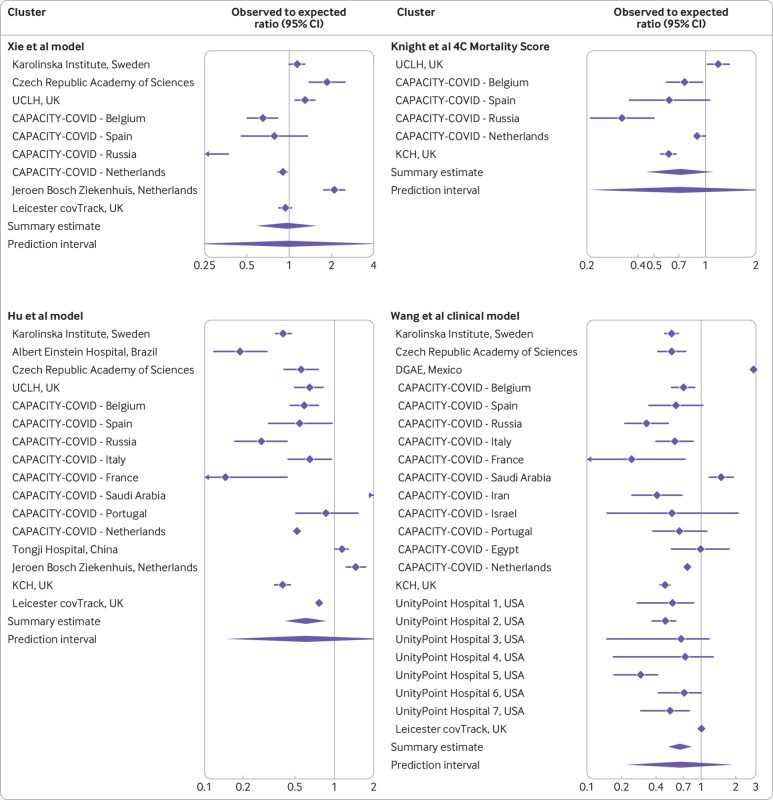
Pooled observed to expected ratio estimates with corresponding 95% confidence interval and approximate 95% prediction interval for four models. Estimates are presented on the log scale. See supplementary file for full data. UCLH=University College London; DGAE=General Directorate of Epidemiology; KCH=King’s College Hospital

The O:E ratio of the Xie model was the closest to 1, with a meta-analysis summary estimate of 0.96 (95% confidence interval 0.59 to 1.55, 95% prediction interval 0.21 to 4.28, fig 4), indicating on average the number of predicted deaths was in agreement with the number of observed deaths. However, the relatively wide 95% confidence interval and 95% prediction interval indicate some heterogeneity. The 4C Mortality Score attained an O:E ratio of 0.71 (0.45 to 1.11, 95% prediction interval 0.21 to 2.39, fig 4). The Hu model attained an O:E ratio of 0.61 (0.42 to 0.87, 95% prediction interval 0.15 to 2.48, fig 4). The Wang clinical model attained an O:E ratio of 0.65 (0.52 to 0.82, 95% prediction interval 0.23 to 1.89, fig 4). Supplementary figure S2 shows the O:E ratios of the other models and supplementary table S10 the calibration-in-the-large values.

### Calibration: slope

Supplementary material D figures S3 and S4 show the forest plots for all calibration slopes. The estimate for the calibration slope was the closest to 1 for the 4C Mortality Score (1.22, 95% confidence interval 0.92 to 1.52, 95% prediction interval 0.63 to 1.80). The Wang clinical model had a calibration slope of 0.50 (0.44 to 0.56, 95% prediction interval 0.34 to 0.66). The calibration slope for the Xie model was 0.45 (0.27 to 0.63, 95% prediction interval −0.07 to 0.96) and for the Hu model was 0.32 (0.15 to 0.49, 95% prediction interval −0.34 to 0.98). Supplementary material D presents details of the remaining models that were estimated.

### Sensitivity analyses—meta-regression

In the meta-regression, where all performance estimates were regressed on the country and the prediction model, the point estimate of the discrimination was the highest for the 4C Mortality Score (reference) and lowest for the Wang laboratory model. Country wise, the point estimate was highest in Israel and lowest in Mexico (see supplementary material D for point estimates and supplementary table S11 for predicted C statistics for each country).

The results for the predicted O:E ratio were less straightforward, as the predicted values for each model except the Wang laboratory model (see supplementary table S12) were greater than 1 in some countries and smaller than 1 in other countries. Similarly, this was the case for the predicted values of the calibration slopes for all models (see supplementary table S13). This implied that none of the included models were well calibrated to the data from all included countries. Supplementary table S14 shows the predicted calibration-in-the-large estimates.

## Discussion

In our individual participant data meta-analysis we found that previously identified prediction models varied in their ability to discriminate between those patients admitted to hospital with covid-19 who will die and those who will survive. The 4C Mortality Score, the Wang clinical model, and the Xie model achieved the highest discrimination on average in our study and could therefore serve as starting points for implementation in clinical practice. The 4C Mortality Score could only be validated in six clusters, which might indicate limited usefulness in clinical practice. Whereas the discrimination of both Wang models and the Xie model was lower than in their respective development studies, the discrimination of the 4C Mortality Score was similar to the estimates in its development study.

Although the summary estimates of discrimination performance are rather precise owing to the large number of included patients, some are prone to substantial between cluster heterogeneity. Discrimination varied greatly across hospitals and countries for all models, but least for the 4C Mortality Score. For some models the 95% prediction interval of the C statistic included 0.5, which implies that in some countries these models might not be able to discriminate between patients with covid-19 who survive or die during hospital admission.

All models were prone to calibration issues. Most models tended to over-predict mortality on average, meaning that the actual death count was lower than predicted. The Xie model achieved O:E ratios closest to 1, but this model’s predicted risks were often too extreme: too high for high risk patients and too low for low risk patients, as quantified by the calibration slope, which was less than 1. The calibration slope was closest to 1 for the 4C Mortality Score, and this was the only model for which the 95% confidence interval included 1. All other summary calibration slopes were less than 1. This could be due to overfitting in the model development process. All the models were prone to substantial between cluster heterogeneity. This implies that local revisions (such as country specific or even centre specific intercepts) are likely necessary to ensure that risk predictions are sufficiently accurate.

Implementing existing covid-19 models in routine care is challenging because the evolution and management of SARS-CoV-2 and the consequences of changes to the virus over time and across geographical areas. In addition, the studied models were developed and validated using data collected during periods of the pandemic, and general practice might have subsequently changed. As a result, baseline risk estimates of existing prediction models (eg, the intercept term) might have less generalisability than anticipated and might require regular updating, as shown in this meta-analysis. As predictor effects might also change over time or geographical region, a subsequent step might be to update these as well.^40^ Since most data originate from electronic health record databases, hospital registries offer a promising source for dynamic updating of covid-19 related prediction models.^41^ ^42^ ^43^ As data from new individuals become available, the prognostic models should be updated, as well as their performance in external validation sets.^41^ ^42^ ^43^

### Limitations of this meta-analysis

All the models we considered were developed and validated using data from the first waves of the covid-19 pandemic, up to April 2021, mostly before vaccination was implemented widely. Since the gathering of data, treatments for patients with covid-19 have improved and new options have been introduced. These changes are likely to reduce the overall risk of short term mortality in patients with covid-19. Prediction models for covid-19 for which adequate calibration has previously been established may therefore still yield inaccurate predictions in contemporary clinical practice. This further highlights the need for continual validation and updates.^43^

An additional concern is that prediction models are typically used to decide on treatment strategies but do not indicate to what extent patients benefit from individualised treatment decisions. Although patients at high risk of death could be prioritised for receiving intensive care, it would be more practical to identify those patients who are most likely to benefit from such care. This individualised approach towards patient management requires models to predict (counterfactual) patient outcomes for all relevant treatment strategies, which is not straightforward.^44^ ^45^ These predictions of patients’ absolute risk reduction require estimation of the patients’ short term risk of mortality with and without treatment, which might require the estimation of treatment effects that differ by patient.^45^

As variants of the disease emerge, new treatments are developed, and the disease is better managed, predictor effects and the incidence of mortality due to covid-19 may vary, thereby potentially limiting the predictive performance of the models we investigated.

We only considered models for predicting mortality in patients with covid-19 admitted to hospital, as outcomes such as clinical deterioration might increase the risk of heterogeneity from variation in measurements and differences in definitions. Mortality, however, is commonly recorded in electronic healthcare systems, with limited risk for misclassification. Furthermore, it is an important outcome that is often considered in decision making.

We had to use the reported nomograms to recover the intercepts for two prediction models from one group.^31^ ^32^ Ideally, authors would have adhered to the TRIPOD guidelines, which would have facilitated the evaluation of their models.

### Conclusion

In this large international study, we found considerable heterogeneity in the performance of the prognostic models for predicting short term mortality in patients admitted to hospital with covid-19 across countries. Caution is therefore needed in applying these tools for clinical decision making in each of these countries. On average, the observed number of deaths was closest to the predicted number of deaths by the Xie model. The 4C Mortality Score and Wang clinical model showed the highest discriminative ability compared with the other validated models. Although they appear most promising, local and dynamic adjustments (intercept and slope updates) are needed before implementation in routine care. The usefulness of the 4C Mortality Score may be affected by the limited availability of the predictor variables.

**What is already known on this topic**

Numerous prognostic models for predicting short term mortality in patients admitted to hospital with covid-19 have been published
These models need to be compared head-to-head in external patient data

**What this study adds**

On average, the 4C Mortality Score by Knight et al and the clinical model by Wang et al showed the highest discriminative ability to predict short term mortality in patients admitted to hospital with covid-19
In terms of calibration, all models require local updating before implementation in new countries or centres

## Data Availability

The data from Tongji Hospital, China that support the findings of this study are available from https://github.com/HAIRLAB/Pre_Surv_COVID_19. Data collected within CAPACITY-COVID is available on reasonable request (see https://capacity-covid.eu/for-professionals/). Data for the CovidRetro study are available on request from MM or the secretariat of the Institute of Microbiology of the Czech Academy of Sciences (contact via mbu@biomed.cas.cz) for researchers who meet the criteria for access to confidential data. The data are not publicly available owing to privacy restrictions imposed by the ethical committee of General University Hospital in Prague and the GDPR regulation of the European Union. We can arrange to run any analytical code locally and share the results, provided the code and the results do not reveal personal information. The remaining data that support the findings of this study are not publicly available.

## Web extra

Extra material supplied by authors

## Notes

Contributors: FvR, JD, MvS, TT, KM, VdJ, TD, BVC, and LW were responsible for the systematic review and design of the study. VdJ and TD were responsible for the statistical analysis plan and R code. FWA, OB-C, VC, RZR, FS, YY, TT, PN, PH, SK, RK, ML, RKG, MN, LFCM, AB, CAPACITY-COVID consortium (see supplementary material E), and CovidRetro collaboration (see supplementary material F) were responsible for primary data collection. RZR, DF, MM, PH, RKG, RN, PN, MN, and ML were responsible for the primary data analysis. RZR and SKK were responsible for the meta-analysis. VdJ and RZR were responsible for the sensitivity analysis. VDJ and RZR were responsible for the initial draft of the manuscript. TD, TT, TLN, ML, FWA, LM, JD, BVC, LW, and KM revised the initial draft. RZR was responsible for the supplementary material on data and results (supplementary material A and D). VdJ and TT were responsible for the supplementary material on models (B). All authors contributed to the critical revision of the manuscript, approved the final version of the manuscript and agree to be accountable for the content. VdJ and RZR contributed equally. VdJ, TD, and KM are the guarantors of this manuscript.

The manuscript’s guarantors (VdJ, TD, and KM) affirm that the manuscript is an honest, accurate, and transparent account of the study being reported; that no important aspects of the study have been omitted; and that any discrepancies from the study as originally planned (and, if relevant, registered) have been explained. All authors had access to statistical reports and tables. Authors did not have access to all data, for privacy, ethical and/or legal reasons. Authors listed under “Primary data collection” in the contributorship section had access to data and take responsibility for the integrity of the data. Authors listed under the analysis bullets in the contributorship section take responsibility for the accuracy of the respective data analyses. The corresponding author attests that all listed authors meet authorship criteria and that no others meeting the criteria have been omitted.

Dissemination to participants and related patient and public communities: We plan to share the results of this study on multiple social media platforms, including Twitter and LinkedIn. Copies of the manuscript will be sent to contributing centres, as well as being shared on the ReCoDID (www.recodid.eu ) and COVID-PRECISE (www.covprecise.org ) websites.
