# Supplementary material for: Clinical prediction models for mortality in patients with covid-19: external validation and individual participant data meta-analysis
Source: BMJ. 2022 Jul 12;378:e069881. doi: 10.1136/bmj-2021-069881 (PMC9273913; doi:10.1136/bmj-2021-069881)
Supplement: Supplementary file 1 — Supplementary information: additional material, A-J [file jonv069881.ww.pdf]

Supplementary Material A - Individual cluster data description and statistical analysis

Data quality check

Data sources were screened before analysis to ensure they met quality requirements. If any quality aspect was unclear, owners of the data were contacted to obtain more information. If the owners did not respond or could not provide sufficient detail, the data source was discarded. For a data source to meet quality requirements, it had to be confirmed that the data only included hospital patients, information on major treatment received by patients was available, (laboratory) measurements had been taken within 24 hours of admission, the outcome was defined clearly, and whether the data was raw or if any analysis had already been conducted. In particular, data in which cases with missing values were systematically excluded or for which missingness had been handled through various means of imputation could not be used and was discarded.

Karolinska Institute – Sweden

Results were shared with us by the Karolinska Institute, Stockholm, Sweden. The data used contained information on 1670 patients recruited between 27 Feb 2020 and 01 Sep 2020. All hospital patients were screened for inclusion. Patients were not included in the analysis if they were under 18 years of age, positive for other viruses on top of being Covid-19 positive, or had already tested positive for Covid-19 in the last 90 days. All patients were followed for at least 30 days. Patients were attributed the “dead” outcome if they died during their hospital stay, which was considered all-cause in-hospital mortality, or if they had died within less than 2 days after being discharged due to administrative delays.

Albert Einstein Hospital – Brazil

Individual patient data was shared with us by the Albert Einstein Israelite Hospital, São Paulo, Brasil. The data contained information on 453 patients admitted between 27 Feb 2020 and 25 Jun 2020. All hospital patients were screened for inclusion. The only inclusion criteria was a positive PCR test for Covid-19, no exclusion criteria were specified. Patients were followed until they died or were discharged. Patients that were labeled as discharged alive or discharged cured were both treated as alive during the analysis. There was a relatively high number of readmissions, with 43 patients being admitted more than once during the recruitment period. For these patients, only the results of their first admission were used during analysis.

Multiple hospitals (unspecified) – Czech Republic

Results were shared from 10 hospitals across Czech Republic. The name and location of the hospitals were not disclosed for anonymity reasons. In total, 213 patients were included in the analysis, recruited between 03 Mar 2020 and 12 Oct 2020. All hospital patients were screened for inclusion. Patients were excluded if they were under 18 years old or if they were already taking part in a drug clinical trial. Patients were followed until they were discharged, dead, or transfered hospitals. Those that were discharged or transferred were

considered alive. At least half of the patients were treated with either hydroxychloroquine, azithromycin, favipiravir, convalescent plasma, dexamethasone, or remdesivir, usually a combination. However, because the effect of the aforementioned drugs was reported to be weak, it was considered acceptable to include results of these patients in our analysis. Full details and analysis of the dataset is available at:

Modrák M, Bürkner P-C, Sieger T, Slisz T, Vašáková M, Mesežnikov G, et al. Disease progression of 213 patients hospitalized with Covid-19 in the Czech Republic in March–October 2020: An exploratory analysis. PLOS ONE. 2021 okt;16(10):e0245103. https://journals.plos.org/plosone/article?id=10.1371/journal.pone.0245103

UCLH – United Kingdom

Data was shared by the University College Hospital London, London, United Kingdom. This data included 411 patients recruited between 01 Feb 2020 and 30 Apr 2020. All hospital patients were screened for inclusion. Patients were excluded if they were under 18 years old or if their positive Covid-19 test was obtained more than 5 days after admission. Patients were followed for at least 30 days, including after being discharged. It is estimated that about a third of patients in this dataset were part of the development population of the 4C Mortality score, but it was not possible to identify where the overlap was.

Directorate General of Epidemiology, Secretariat of Health – Mexico City

The data provided by the Directorate General of Epidemiology included 28176 patients from virtually all hospitals across Mexico. Patients were recruited between 01 Mar 2020 and 16 Apr 2020. All hospital patients were screened for inclusion. Patients of all ages were included, even babies under 1 year of age. Follow-up lasted at least 30 days. Patients that were discharged were considered alive for the analysis, while those that died of all-cause mortality were considered dead from Covid-19.

CAPACITY-COVID

CAPACITY-COVID contained data from multiple sites across multiple countries. At the time of analysis, data from 11 countries and 62 sites was available, for a total of 6392 patients (Table S1). Recruitment dates varied per country. Only patients above 18 years old were included. Patients that were discharged alive, transferred to another facility, or on palliative discharge were all considered alive. Analysis was performed per country. CAPACITY-COVID entries were not always correct or complete. When values were deemed incorrect, such as negative lymphocyte counts, an oxygen saturation % above 100, or a Glasgow Coma Scale below 3 or above 15, they were censored. For laboratory measurements, if the unit could not reliably be identified, the measurement was removed. Extreme outliers were also removed. Each site used their own inclusion process (Table S1).

Table S1: Number of sites and patients per country within CAPACITY-COVID dataset

Country	Number of sites	Number of patients	Site inclusion process (number of patients)
Belgium	5	221	2 sites included all admitted patients (148), 3 sites only included cardiology patients (73)
Egypt	2	45	1 site included all admitted patients (44), 1 site only included cardiology patients (1)
France	1	46	All admitted patients were included
Iran	1	90	Only cardiology patients were included
Israel	1	25	All admitted patients were included
Italy	1	106	Only cardiology patients were included
Netherlands	45	5100	37 sites included all admitted patients (4426), 1 site included all patients excluding ICU patients (124), 7 sites only included cardiology patients (550)
Portugal	2	44	1 site included all patients (27), 1 site only included ICU patients (17)
Russian Federation	1	278	All admitted patients were included
Saudi Arabia	1	389	All admitted patients were included
Spain	2	47	All admitted patients were included

**Variable**	**Missingness (%)**
Age	1.58
Sex	0.66
Diabetes (type 2)	3.05
Diabetes (all types)	3.07
Obesity	29.07
Pneumonia	84.04
Chronic kidney disease	2.28
COPD	3.10

Immunosuppression	86.15
LDH	92.60
Lymphocyte count	20.70
SpO2 (all measurement types)	10.37
SpO2 (room air measurement only)	47.36
CRP	8.26
D-Dimer	77.93
Chronic lung disease	71.10
Malignancy	79.96
Hypertension	3.85
Heart disease	2.30
Chronic heart disease	0.02
Cough	2.39
Dyspnea	1.69
Neutrophil count	92.18
Platelet count	11.64
Creatinine count	11.28
Number of comorbidities	0
Glasgow Coma Scale	95.06
Urea	90.08
Respiratory rate	13.03
Mortality	2.64

Multiple hospitals (unspecified) – China (Wuhan region)

Data was shared from multiple hospitals in the Wuhan region, China. Names and locations of hospitals were kept anonymous. A total of 332 were included, all recruited between 10 Jan 2020 and 18 Feb 2020. All hospital patients were screened for inclusion. Patients were excluded if they were under 18 years old, pregnant, or breastfeeding. Patients were followed until they recovered or until they died.

UnityPoint hospitals – United States (Iowa)

Patient data was shared from 7 UnityPoint hospitals, Iowa, United States. In total, 2577 were included, although some hospitals provided more data than others (see Table 2 in main text). All hospital patients were screened for inclusion. Patients were recruited between 03 Mar 2020 and 31 Jul 2020, and all patients were included regardless of age, including babies under 1 year old. Patients were followed for 30 days, at which point they were simply observed to be alive or dead.

Jeroen Bosch Ziekenhuis – the Netherlands

The Jeroen Bosch Ziekenhuis, 's-Hertogenbosch, the Netherlands shared data of 383 patients. Patients were recruited between 09 Mar 2020 and 29 Dec 2020. All patients were above 18 years old and followed for 30 days. Patients whose death were known but occurred after the 30 days mark were reported as alive and treated as such during the analysis. Some of the laboratory values were capped at low or high values. CRP levels < 3

mg/L, neutrophils levels < 0.1 10^9/L, AST levels < 8 U/L, and eGFR > 90 ml/min were capped. D-Dimer levels were initially capped at > 4 mg/L, but after D-Dimer levels were determined to be an important predictor of prognosis, this value was raised to > 20 mg/L. All capped values were rounded before analysis.

Leicester covTrack – United Kingdom

Data provided by Leicester covTrack was obtained from 3908 patients admitted in the University Hospitals of Leicester, Leicester, United Kingdom between Jan 2020 and Apr 2021. All hospital patients were screened for inclusion. Patients under 18 years of age were excluded and all patients were followed for at least 30 days.

King's College London – United Kingdom

Patient data was shared by the King's College London, London, United Kingdom. A total of 2400 patients were included, but only 2321 were included for the validation of the 4C Score, and 1138 were used to validate the Hu model. All patients were used for the validation of the other models. All hospital patients were screened for inclusion. Patients were recruited between 28 Feb 2020 and 28 Mar 2021. No specific inclusion or exclusion criteria were mentioned. Patients were followed for a duration of at least 30 days.

Supplementary Material B – Additional statistical analysis and prediction models and risk scores

Statistical analysis - Validation

We imputed sporadically missing data 50 times by applying the Multiple Imputation by Chained Equations procedure using the Full Conditional Specification or Joint Modelling (JOMO) within each dataset separately. The imputation models included: the predictors of the prognostic models to be validated, mortality, and the Nelson-Aalen estimate of time to mortality, where available. We did not impute systematically missing variables. For the Bello-Chavolla scoring rule, which yielded a score instead of an absolute risk, we only assessed the discrimination performance. Then, we pooled the estimates of the performance metrics across imputation iterations using Rubin's rules. To warrant the normality assumption, we performed this pooling on the logit scale for the c-statistic and the log scale for the O:E ratio.

Here is the R code used for the imputation process. The first line is a false run, to ensure mice can be run in our dataset. If for example there are too many missing values for a variable or the data has not be cleaned properly, it will be visible at this stage. "data_miss" is the raw data with missing values. The second line is the real imputation process, where the iteration methods are derived from the false run, and the number of imputation runs is set to 50 (m = 50).

```
setup_imp <- mice(data_miss, maxit=0)
data_imp <- mice(data_miss, method = setup_imp$method, m = 50, maxit = 25, printFlag = F)
```

Bello-Chavolla et al. model

Risk score = 3 * age ≥65 years + 1 * diabetes + 5 * diabetes * age < 40 - 6 * age < 40 years + 1 * obesity + 7 * pneumonia + 3 * chronic kidney disease + 1 * chronic obstructive pulmonary disease + 1 * immunosuppression

Bello-Chavolla OY, Bahena-López JP, Antonio-Villa NE, Vargas-Vázquez A, González-Díaz A, Márquez-Salinas A, Fermín-Martínez CA, Naveja JJ, Aguilar-Salinas CA. Predicting mortality due to SARS-CoV-2: A mechanistic score relating obesity and diabetes to COVID-19 outcomes in Mexico. J. Clin. Endocrinol. Metab. 2020; https://doi.org/10.1210/clinem/dgaa346

Xie et al. model

logit(outcome) = 4.559 + 0.047 * age + 0.003 * lactate dehydrogenase (U/L) - 1.094 * lymphocyte count (10^9/L) - 0.098 * SPO2 (%)

Xie J, Hungerford D, Chen H, Abrams ST, Li S, Wang G, Wang Y, Kang H, Bonnett L, Zheng R, Li X, Tong Z, Du B, Qiu H, Toh C-H. Development and external validation of a prognostic multivariable model on admission for hospitalized patients with COVID-19. medRxiv Cold

Spring Harbor Laboratory Press; 2020; : 2020.03.28.20045997
medrxiv.org/content/medrxiv/early/2020/04/07/2020.03.28.20045997.full.pdf

Hu et al. model

logit(outcome) = -4.211 + 0.013 * high-sensitivity c-reactive protein (mg/L) + 0.059 * age + 0.112 * D-Dimer (ug/dL) - 1.984 * lymphocyte count (10^9/L)

Chen X, Liu Z. Early prediction of mortality risk among severe COVID-19 patients using machine learning. medRxiv Cold Spring Harbor Laboratory Press; 2020; : 2020.04.13.20064329

https://www.medrxiv.org/content/10.1101/2020.04.13.20064329v1.full.pdf

Zhang et al. models

DCS model

logit(outcome) = -8.50 + 0.05 * age + 0.31 * male + 1.10 * chronic lung disease + 0.44 * diabetes mellitus + 1.38 * immunocompromised + 0.02 * malignancy + 0.34 * hypertension + 0.68 * heart disease + 1.32 * chronic renal disease

DCSL model

logit(outcome) = -3.20 - 0.87 * male + 0.41 * chronic lung disease + 0.31 * diabetes mellitus + 0.94 * cough + 0.85 * dyspnoea + 0.18 * neutrophil count (10^9/L) - 1.43 * lymphocyte count (10^9/L) - 0.01 * platelet count (10^9/L) + 0.01 * c-reactive protein (mg/L)

Zhang H, Shi T, Wu X, Zhang X, Wang K, Bean D, Dobson R, Teo JT, Sun J, Zhao P, Li C, Dhaliwal K, Wu H, Li Q, Guthrie B. Risk prediction for poor outcome and death in hospital inpatients with COVID-19: derivation in Wuhan, China and external validation in London, UK. medRxiv Cold Spring Harbor Laboratory Press; 2020; : 2020.04.28.20082222.
https://www.medrxiv.org/content/10.1101/2020.04.28.20082222v1.full.pdf

4C Mortality Score

2 * age 50-59 + 4 * age 60-69 + 6 * age 70-79 + 7 * age > 80 + 1 * male + 1 * one comorbidity + 2 * multiple comorbidities + 1 * respiratory rate (breath/minute) 20-29 + 2 * respiratory rate (breaths/minute) ≥ 30 + 2 * oxygen saturation on room air (%) < 92 + 2 * Glasgow coma scale < 15 + urea (mmol/L) 7-14 + 3 * urea (mmol/L) > 14 + 1 * c-reactive protein (mg/L) 50-99 + 2 * c-reactive protein (mg/L) ≥ 100)

Comorbidities used in model: chronic cardiac disease, chronic respiratory disease (excluding asthma), chronic renal disease (eGFR =< 30), liver disease (moderate to severe), dementia, chronic neurological conditions, connective tissue disease, diabetes, AIDS/HIV, malignancy, obesity (clinically defined)

As no estimated risk was available for a score of 0, we used a predicted risk of 0.001%. This is practically zero, but unlike zero does not lead to estimation problems.

4C Mortality Score	Mortality %	Mortality probability
0	0.001	0.00001
1	0.3	0.003
2	0.8	0.008
3	2.3	0.023
4	4.8	0.048
5	7.5	0.075
6	7.8	0.078
7	11.7	0.117
8	14.4	0.144
9	19.2	0.192
10	22.9	0.229
11	26.9	0.269
12	32.9	0.329
13	40.1	0.401
14	44.6	0.446
15	51.6	0.516
16	59.1	0.591
17	66.1	0.661
18	75.8	0.758
19	77.4	0.774
20	82.9	0.829
21	87.5	0.875

Knight SR, Ho A, Pius R, Buchan I, Carson G, Drake TM, Dunning J, Fairfield CJ, Gamble C, Green CA, Gupta R. Risk stratification of patients admitted to hospital with covid-19 using the ISARIC WHO Clinical Characterisation Protocol: development and validation of the 4C Mortality Score. bmj. 2020 Sep 9;370. https://www.bmj.com/content/370/bmj.m3339

Wang et al. models

Clinical model

logit(outcome) = -8.6 + 0.10 * age + 0.60 * history of hypertension + 1.11 * history of coronary heart disease

Laboratory model

logit(outcome) = -8.93 + 0.095 * age - 0.34 * SpO2 (%) + 0.31 * neutrophil count (10^9/L) - 0.67 * lymphocyte count (10^9/L) + 0.039 * high-sensitivity C reactive protein (mg/L) - 0.58 * D-Dimer (ug/mL) + 0.05 * aspartate aminotransferase (U/L) + 0.05 * glomerular filtration rate (ml/min)

Wang K, Zuo P, Liu Y, Zhang M, Zhao X, Xie S, Zhang H, Chen X, Liu C. Clinical and laboratory predictors of in-hospital mortality in patients with COVID-19: a cohort study in Wuhan, China. Clinical infectious diseases. 2020 May 3. https://academic.oup.com/cid/advance-article/doi/10.1093/cid/ciaa538/5828281

Supplementary Material C – TRIPOD Checklist for model validation

Section/Topic		Checklist Item	Page
**Title and abstract**			
Title	1	Identify the study as developing and/or validating a multivariable prediction model, the target population, and the outcome to be predicted.	1
Abstract	2	Provide a summary of objectives, study design, setting, participants, sample size, predictors, outcome, statistical analysis, results, and conclusions.	4
**Introduction**			
Background and objectives	3a	Explain the medical context (including whether diagnostic or prognostic) and rationale for developing or validating the multivariable prediction model, including references to existing models.	5
	3b	Specify the objectives, including whether the study describes the development or validation of the model or both.	5
**Methods**			
Source of data	4a	Describe the study design or source of data (e.g., randomized trial, cohort, or registry data), separately for the development and validation data sets, if applicable.	6-7
	4b	Specify the key study dates, including start of accrual; end of accrual; and, if applicable, end of follow-up.	Table 2
Participants	5a	Specify key elements of the study setting (e.g., primary care, secondary care, general population) including number and location of centres.	6-7; Table 2
	5b	Describe eligibility criteria for participants.	6-7
	5c	Give details of treatments received, if relevant.	N/A
Outcome	6a	Clearly define the outcome that is predicted by the prediction model, including how and when assessed.	7
	6b	Report any actions to blind assessment of the outcome to be predicted.	N/A
Predictors	7a	Clearly define all predictors used in developing or validating the multivariable prediction model, including how and when they were measured.	Table 1
	7b	Report any actions to blind assessment of predictors for the outcome and other predictors.	N/A
Sample size	8	Explain how the study size was arrived at.	7
Missing data	9	Describe how missing data were handled (e.g., complete-case analysis, single imputation, multiple imputation) with details of any imputation method.	7
Statistical analysis methods	10c	For validation, describe how the predictions were calculated.	7, Supplement B
	10d	Specify all measures used to assess model performance and, if relevant, to compare multiple models.	7-8
	10e	Describe any model updating (e.g., recalibration) arising from the validation, if done.	N/A
Risk groups	11	Provide details on how risk groups were created, if done.	N/A
Development vs. validation	12	For validation, identify any differences from the development data in setting, eligibility criteria, outcome, and predictors.	Table 1&2
**Results**			
Participants	13a	Describe the flow of participants through the study, including the number of participants with and without the outcome and, if applicable, a summary of the follow-up time. A diagram may be helpful.	9
	13b	Describe the characteristics of the participants (basic demographics, clinical features, available predictors), including the number of participants with missing data for predictors and outcome.	13-18, Table 2
	13c	For validation, show a comparison with the development data of the distribution of important variables (demographics, predictors and outcome).	Table 1&2
Model performance	16	Report performance measures (with CIs) for the prediction model.	19-20, Fig 3-5, Supplement D
Model-updating	17	If done, report the results from any model updating (i.e., model specification, model performance).	N/A
**Discussion**			
Limitations	18	Discuss any limitations of the study (such as nonrepresentative sample, few events per predictor, missing data).	22
Interpretation	19a	For validation, discuss the results with reference to performance in the development data, and any other validation data.	21
	19b	Give an overall interpretation of the results, considering objectives, limitations, results from similar studies, and other relevant evidence.	20-22
Implications	20	Discuss the potential clinical use of the model and implications for future research.	21-22
**Other information**			
Supplementary information	21	Provide information about the availability of supplementary resources, such as study protocol, Web calculator, and data sets.	Supplement A, B, D
Funding	22	Give the source of funding and the role of the funders for the present study.	24-26

Supplementary Material D - Individual results of each model validation per cluster

Table S2: Individual Results of Wang Clinical model

Cluster	Country	c-statistic (95% CI)	Calibration slope (95% CI)	Calibration-in-the-large (95% CI)	O:E ratio (95% CI)
Karolinska Institutet	Sweden	0.82 (0.79 to 0.85)	0.60 (0.51 to 0.68)	-1.16 (-1.35 to -0.98)	0.13 (0.11 to 0.15)
Czech Republic Academy of Sciences	Czech Republic	0.82 (0.73 to 0.89)	0.80 (0.50 to 1.11)	-1.20 (-1.61 to -0.80)	0.55 (0.41 to 0.73)
UCLH*	United Kingdom	0.74 (0.69 to 0.79)	0.49 (0.35 to 0.62)	-5.97 (-6.26 to -5.69)	0.31 (0.26 to 0.36)
General Directorate of Epidemiology of the Mexican Ministry of Health (DGAE)	Mexico	0.67 (0.67 to 0.68)	0.39 (0.37 to 0.40)	2.16 (2.13 to 2.19)	2.90 (2.86 to 2.94)
CAPACITY-COVID	Belgium	0.73 (0.64 to 0.80)	0.47 (0.26 to 0.68)	-0.75 (-1.12 to -0.37)	0.70 (0.55 to 0.88)
	Spain	0.93 (0.77 to 0.98)	2.23 (0.48 to 3.98)	-1.00 (-1.80 to -0.19)	0.60 (0.35 to 1.04)
	Russia	0.85 (0.76 to 0.90)	0.75 (0.42 to 1.07)	-1.75 (-2.28 to -1.22)	0.33 (0.22 to 0.51)
	Italy	0.74 (0.62 to 0.83)	0.68 (0.26 to 1.10)	-0.99 (-1.52 to -0.46)	0.58 (0.40 to 0.85)
	France	0.76 (0.47 to 0.92)	0.46 (-0.13 to 1.04)	-1.03 (-1.95 to -0.11)	0.25 (0.08 to 0.72)
	Saudi Arabia	0.73 (0.66 to 0.78)	0.46 (0.28 to 0.63)	0.65 (0.30 to 1.00)	1.49 (1.17 to 1.90)

	Iran	0.75 (0.58 to 0.86)	0.72 (0.19 to 1.25)	-1.53 (-2.19 to -0.88)	0.41 (0.25 to 0.68)
	Israel	0.98 (0.73 to 1.00)	7.99 (-6.13 to 22.12)	-0.83 (-2.41 to 0.75)	0.56 (0.15 to 2.12)
	Portugal	0.53 (0.32 to 0.73)	0.02 (-0.44 to 0.48)	-0.84 (-1.65 to -0.03)	0.65 (0.38 to 1.11)
	Egypt	0.69 (0.45 to 0.85)	0.35 (-0.15 to 0.85)	-0.02 (-0.85 to 0.81)	0.99 (0.55 to 1.77)
	Netherlands	0.71 (0.70 to 0.73)	0.52 (0.47 to 0.57)	-0.50 (-0.58 to -0.42)	0.75 (0.71 to 0.80)
King's College London	United Kingdom	0.75 (0.72 to 0.78)	0.43 (0.37 to 0.50)	-1.41 (-1.56 to -1.27)	0.48 (0.43 to 0.54)
UnityPoint Hospitals	United States (Iowa)	0.89 (0.84 to 0.94)	0.72 (0.40 to 0.50)	-0.85 (-1.47 to -0.24)	0.56 (0.28 to 0.85)
		0.77 (0.71 to 0.82)	0.42 (0.30 to 0.54)	-1.24 (-1.53 to -0.95)	0.48 (0.37 to 0.60)
		0.90 (0.77 to 1.00)	0.97 (0.30 to 1.63)	-0.61 (-1.56 to 0.34)	0.67 (0.15 to 1.18)
		0.62 (0.46 to 0.78)	0.14 (-0.22 to 0.51)	-0.54 (-1.55 to 0.46)	0.72 (0.17 to 1.27)
		0.82 (0.77 to 0.87)	0.57 (0.32 to 0.82)	-1.81 (-2.28 to -1.34)	0.30 (0.18 to 0.42)
		0.81 (0.73 to 0.90)	0.61 (0.35 to 0.87)	-0.48 (-0.96 to 0.00)	0.71 (0.42 to 1.00)
		0.80 (0.73 to 0.88)	0.44 (0.21 to 0.66)	-1.07 (-1.65 to -0.49)	0.54 (0.30 to 0.78)
Leicester covTrack	United Kingdom	0.78 (0.76 to 0.79)	0.55 (0.50 to 0.59)	0.00 (-0.09 to 0.09)	1.00 (0.95 to 1.05)

* This group used a different intercept for the Wang et al. clinical model, therefore it was omitted from the meta-analyses of the O:E ratio and the calibration-in-the-large.

Table S3: Individual Result of Hu model

Cluster	Country	c-statistic (95% CI)	Calibration slope (95% CI)	Calibration-in-the-large (95% CI)	O:E (95% CI)
Karolinska Institutet	Sweden	0.77 (0.73 to 0.81)	0.25 (0.10 to 0.40)	-1.98 (-2.19 to -1.76)	0.41 (0.36 to 0.47)
Albert Einstein Hospital	Brazil	0.83 (0.69 to 0.92)	0.60 (0.35 to 0.86)	-2.82 (-3.41 to -2.23)	0.19 (0.12 to 0.30)
Czech Republic Academy of Sciences	Czech Republic	0.70 (0.58 to 0.80)	0.08 (-0.03 to 0.19)	-1.42 (-1.77 to -1.07)	0.56 (0.42 to 0.74)
UCLH	United Kingdom	0.59 (0.49 to 0.69)	0.13 (-0.02 to 0.27)	-1.20 (-1.67 to -0.73)	0.64 (0.50 to 0.82)
CAPACITY-COVID	Belgium	0.65 (0.53 to 0.75)	0.03 (-0.08 to 0.13)	-1.23 (-1.65 to -0.80)	0.59 (0.46 to 0.75)
	Spain	0.76 (0.55 to 0.89)	0.75 (-0.17 to 0.61)	-0.40 (-1.19 to 0.38)	0.55 (0.31 to 0.96)
	Russia	0.89 (0.77 to 0.95)	0.30 (-0.02 to 0.61)	-2.90 (-4.31 to -1.49)	0.27 (0.17 to 0.44)
	Italy	0.60 (0.44 to 0.75)	0.05 (-0.04 to 0.15)	-1.60 (-2.81 to -0.39)	0.65 (0.44 to 0.95)
	France	0.78 (0.49 to 0.93)	0.26 (-0.22 to 0.74)	-2.28 (-3.31 to -1.25)	0.15 (0.05 to 0.43)
	Saudi Arabia	0.60 (0.49 to 0.69)	0.02 (-0.06 to 0.10)	1.42 (1.05 to 1.79)	2.54 (1.96 to 3.29)
	Portugal	0.63 (0.41 to 0.81)	0.33 (-0.14 to 0.80)	-0.27 (-1.12 to 0.58)	0.87 (0.50 to 1.50)

	Netherlands	0.70 (0.68 to 0.72)	0.54 (0.41 to 0.67)	-0.17 (-0.28 to -0.05)	0.51 (0.49 to 0.54)
Huazhong University of Science and Technology (HUST)	China	0.95 (0.93 to 0.97)	1.30 (1.02 to 1.59)	0.54 (0.22 to 0.87)	1.15 (1.02 to 1.29)
Jeroen Bosch Ziekenhuis	Netherlands	0.74 (0.61 to 0.83)	0.46 (0.03 to 0.89)	1.14 (0.55 to 1.72)	1.46 (1.23 to 1.72)
King’s College London	United Kingdom	0.64 (0.60 to 0.69)	0.21 (0.13 to 0.28)	-1.98 (-2.18 to -1.79)	0.41 (0.36 to 0.46)
Leicester covTrack	United Kingdom	0.75 (0.73 to 0.78)	0.35 (0.18 to 0.53)	-0.67 (-0.77 to -0.57)	0.76 (0.72 to 0.80)

Table S4: Individual Results of Bello-Chavolla model

Cluster	Country	c-statistic (95% CI)	Calibration slope (95% CI)	Calibration-in-the-large (95% CI)	O:E (95% CI)
UCLH	United Kingdom	0.68 (0.61 to 0.74)			
General Directorate of Epidemiology of the Mexican Ministry of Health (DGAE)	Mexico	0.62 (0.62 to 0.63)			
CAPACITY-COVID	Belgium	0.78 (0.67 to 0.87)			
	Russia	0.78 (0.63 to 0.88)			
	Iran	0.71 (0.53 to 0.84)			
	Netherlands	0.70 (0.67 to 0.72)			
King’s College London	United Kingdom	0.65 (0.62 to 0.68)			

Table S5: Individual Result of Xie model

Cluster	Country	c-statistic (95% CI)	Calibration slope (95% CI)	Calibration-in-the-large (95% CI)	O:E (95% CI)
Karolinska Institute	Sweden	0.81 (0.77 to 0.84)	0.59 (0.34 to 0.85)	0.20 (0.01 to 0.35)	1.13 (0.99 to 1.30)
Czech Republic Academy of Sciences	Czech Republic	0.75 (0.62 to 0.84)	0.20 (-0.03 to 0.42)	0.88 (0.47 to 1.28)	1.85 (1.38 to 2.47)
UCLH	United Kingdom	0.71 (0.64 to 0.77)	0.59 (0.35 to 0.83)	0.44 (0.18 to 0.71)	1.29 (1.09 to 1.52)
CAPACITY-COVID	Belgium	0.61 (0.49 to 0.72)	0.03 (-0.11 to 0.17)	-0.97 (-1.44 to -0.51)	0.65 (0.50 to 0.83)
	Spain	0.73 (-0.50 to 0.88)	0.75 (-0.17 to 1.67)	-0.40 (-1.19 to 0.38)	0.78 (0.45 to 1.35)
	Russia	0.89 (0.81 to 0.94)	0.71 (0.30 to 1.11)	-2.54 (-3.23 to -1.86)	0.24 (0.15 to 0.37)
	Netherlands	0.73 (0.69 to 0.76)	0.54 (0.41 to 0.67)	-0.17 (-0.28 to -0.05)	0.89 (0.83 to 0.96)
Jeroen Bosch Ziekenhuis	Netherlands	0.72 (0.61 to 0.80)	0.56 (0.10 to 1.02)	1.70 (1.18 to 2.21)	2.08 (1.76 to 2.46)
Leicester covTrack	United Kingdom	0.76 (0.71 to 0.8)	0.51 (0.29 to 0.72)	-0.15 (-0.37 to 0.07)	0.93 (0.84 to 1.04)

Table S6: Individual Result of Zhang DCS model

Cluster	Country	c-statistic (95% CI)	Calibration slope (95% CI)	Calibration-in-the-large (95% CI)	O:E (95% CI)
General Directorate of Epidemiology of the Mexican Ministry of Health (DGAE)	Mexico	0.66 (0.66 to 0.67)	0.48 (0.46 to 0.50)	3.19 (3.17 to 3.22)	7.81 (7.71 to 7.92)
CAPACITY-COVID	Spain	0.90 (0.72 to 0.97)	1.29 (0.43 to 2.15)	0.97 (0.09 to 1.84)	1.74 (1.00 to 3.03)
	Saudi Arabia	0.70 (0.63 to 0.77)	0.50 (0.31 to 0.70)	1.85 (1.54 to 2.16)	3.11 (2.42 to 4.00)
	Netherlands	0.65 (0.63 to 0.68)	0.32 (0.29 to 0.38)	0.22 (0.11 to 0.33)	1.12 (1.04 to 1.19)
King's College London	United Kingdom	0.73 (0.70 to 0.75)	0.36 (0.29 to 0.42)	1.57 (1.41 to 1.72)	2.62 (2.35 to 2.91)
Leicester covTrack	United Kingdom	0.73 (0.71 to 0.75)	0.47 (0.41 to 0.54)	1.53 (1.42 to 1.63)	2.28 (2.12 to 2.45)

Table S7: Individual Results of Zhang DCSL model

Cluster	Country	c-statistic (95% CI)	Slope (95% CI)	Calibration-in-the-large (95% CI)	O:E ratio (95% CI)
CAPACITY-COVID	Belgium	0.59 (0.47 to 0.69)	0.04 (-0.08 to 0.15)	0.78 (0.36 to 1.20)	1.48 (1.15 to 1.90)
	Spain	0.57 (0.35 to 0.77)	0.03 (-0.22 to 0.27)	0.96 (-0.51 to 2.43)	1.40 (0.71 to 2.77)
	Russia	0.89 (0.77 to 0.95)	1.07 (0.55 to 1.59)	0.21 (-0.33 to 0.75)	1.16 (0.75 to 1.81)
	Netherlands	0.62 (0.60 to 0.65)	0.17 (0.12 to 0.22)	0.41 (0.30 to 0.53)	1.21 (1.13 to 1.30)
King’s College London	United Kingdom	0.71 (0.68 to 0.74)	0.34 (0.28 to 0.41)	0.98 (0.83 to 1.13)	1.80 (1.62 to 2.00)
Leicester covTrack	United Kingdom	0.63 (0.60 to 0.65)	0.16 (0.06 to 0.25)	1.11 (1.00 to 1.22)	1.69 (1.60 to 1.80)

Table S8: Individual Results of 4C Score

Cluster	Country	c-statistic (95% CI)	Calibration slope (95% CI)	Calibration-in-the-large (95% CI)	O:E ratio (95% CI)
Karolinska Institutet*	Sweden	0.87 (0.84 to 0.89)	0.38 (0.32 to 0.45)	-2.23 (-2.66 to -1.79)	0.52 (0.44 to 0.60)
UCLH	United Kingdom	0.81 (0.76 to 0.85)	1.47 (1.12 to 1.81)	0.29 (0.05 to 0.52)	1.20 (1.03 to 1.40)
CAPACITY-COVID	Belgium	0.81 (0.72 to 0.87)	1.31 (0.77 to 1.85)	-0.46 (-0.81 to -0.12)	0.75 (0.59 to 0.96)
	Spain	0.86 (0.66 to 0.95)	1.65 (0.08 to 3.22)	-0.86 (-1.66 to -0.07)	0.61 (0.36 to 1.06)
	Russia	0.90 (0.82 to 0.94)	2.04 (1.09 to 2.99)	-1.47 (-1.96 to -0.98)	0.32 (0.21 to 0.50)
	Netherlands	0.77 (0.75 to 0.79)	1.05 (0.91 to 1.19)	-0.13 (-0.21 to -0.05)	0.90 (0.85 to 1.00)
King's College London	United Kingdom	0.788 (0.76 to 0.82)	1.03 (0.88 to 1.19)	-0.69 (-0.82 to -0.56)	0.61 (0.55 to 0.68)

* This group used a different interpretation of the 4C rule. Their results are included in this table but were not used during the meta-analysis of the 4C Score.

Table S9: Individual Results of Wang Laboratory model

Cluster	Country	c-statistic (95% CI)	Calibration slope (95% CI)	Calibration-in-the-large (95% CI)	O:E ratio (95% CI)
Karolinska Institutet	Sweden	0.69 (0.64 to 0.73)	0.09 (0.06 to 0.12)	20.8 (20.4 to 21.2)	6139 (1579 to 23879)
Jeroen Bosch Ziekenhuis	Netherlands	0.67 (0.54 to 0.78)	0.12 (0.01 to 0.23)	24.80 (23.56 to 26.04)	271.83 (29.39 to 2514.01)
Leicester covTrack	United Kingdom	0.65 (0.61 to 0.69)	0.10 (0.06 to 0.14)	22.19 (21.32 to 23.07)	273.20 (60.71 to 1229.50)

Forest plots of the Bello-Chavolla, Zhang DCS, Zhang DCSL, Wang laboratory models

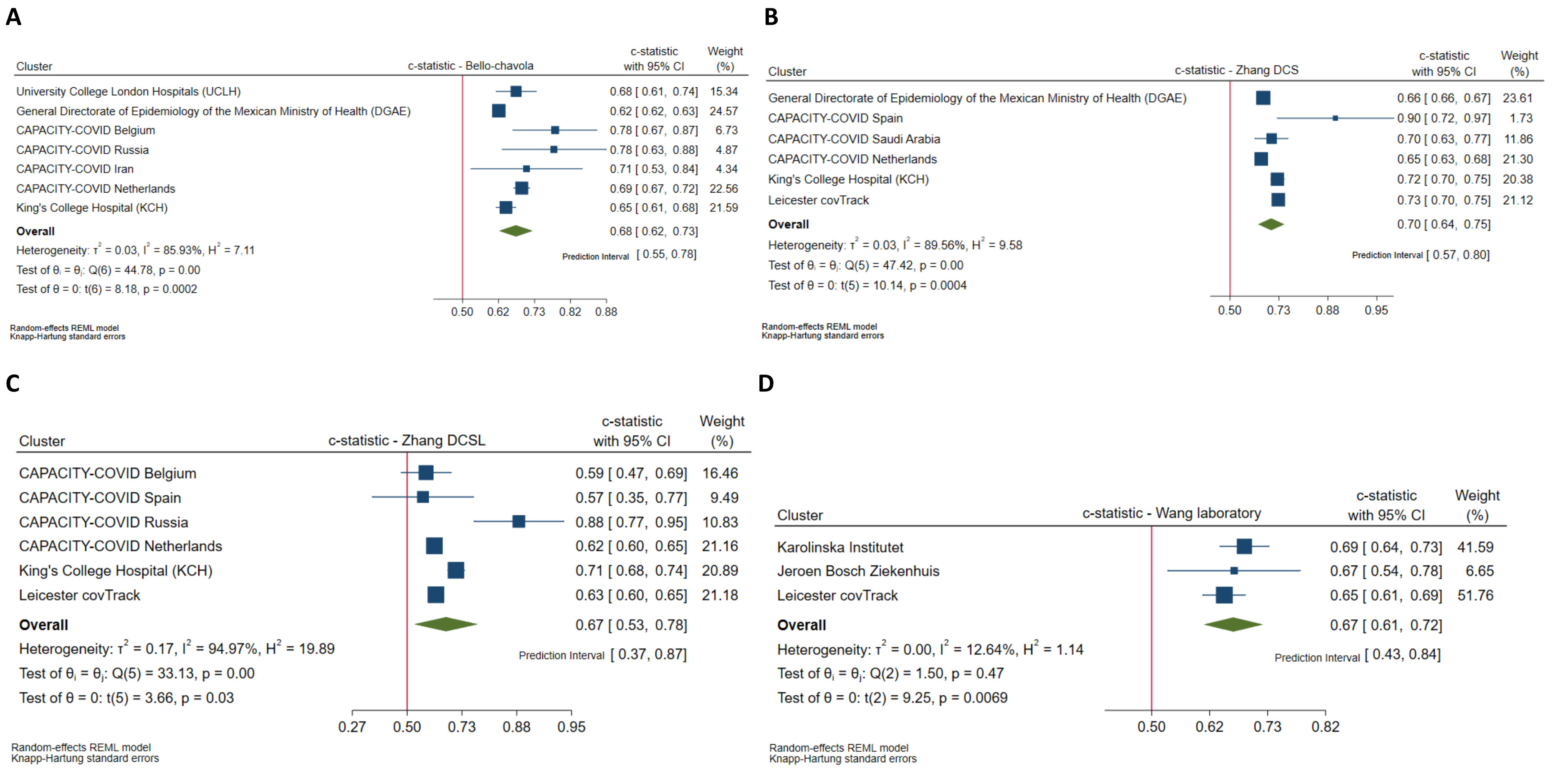

Figure S1: Pooled c-statistic estimates with corresponding 95% CI and approximate 95% PI. A: Bello-Chavolla model. B: Zhang DCS model. C: Zhang DCSL model. D: Wang laboratory model. Clusters marked with a * are part of CAPACITY-COVID. The laboratory model by Wang et al had a c-statistic of 0.98 (0.92 to 0.99) in the development data, and 0.88 (0.75 to 0.96) in the validation data in the original publication. The score by Bello-Chavolla et al had a c-statistic of 0.81 in the development data, and 0.83 in the validation data in the original publication. The DCS model by Zhang et al had a c-statistic of 0.79 in the development data in the original publication. The DCSL model by Zhang et al had a c-statistic of 0.89 in the development data in the original publication.

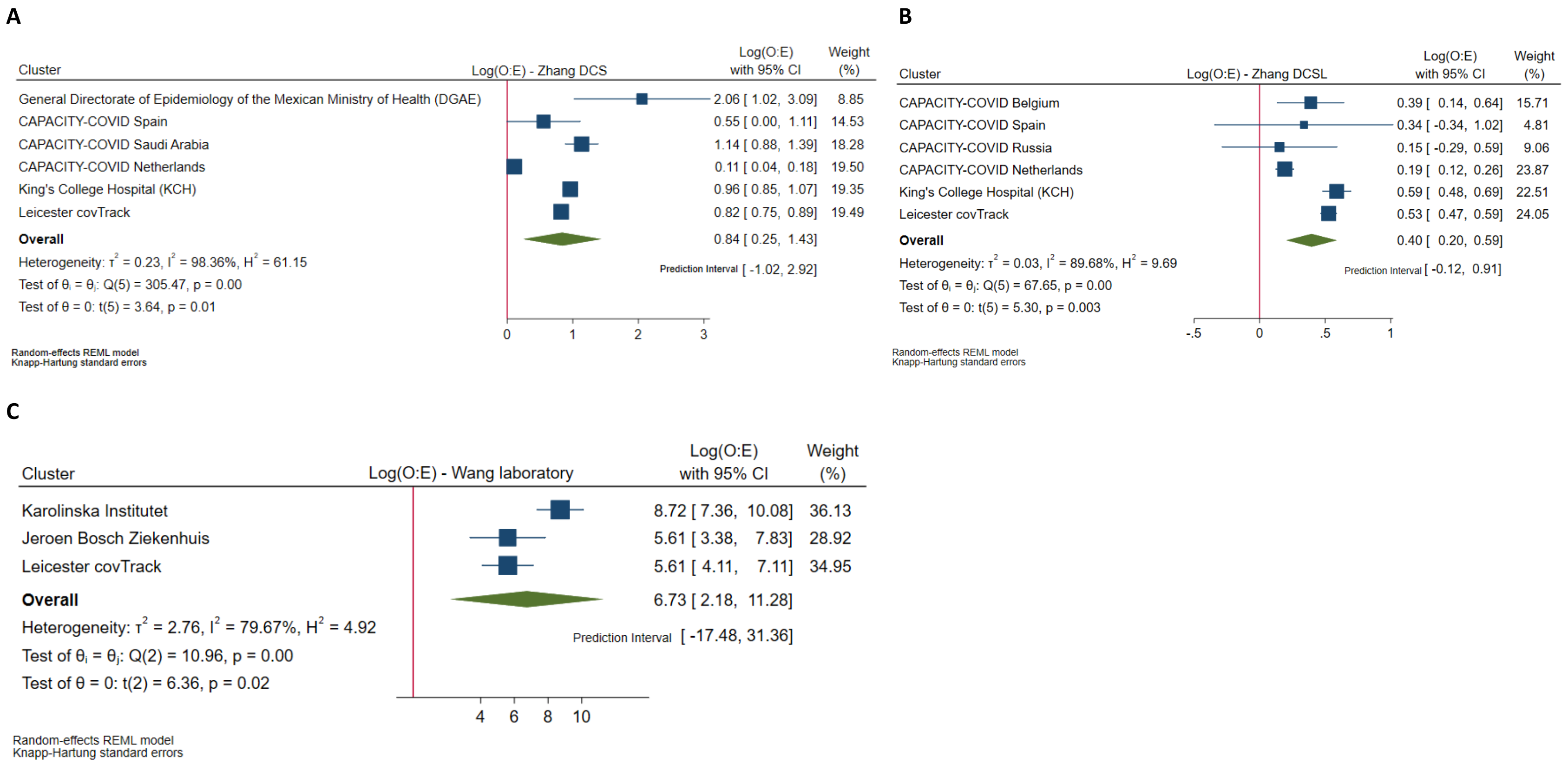

Figure S2: Pooled O:E ratio estimates with corresponding 95 % CI and approximate 95% PI. A: Zhang DCS model. B: Zhang DCSL model. C: Wang laboratory model.

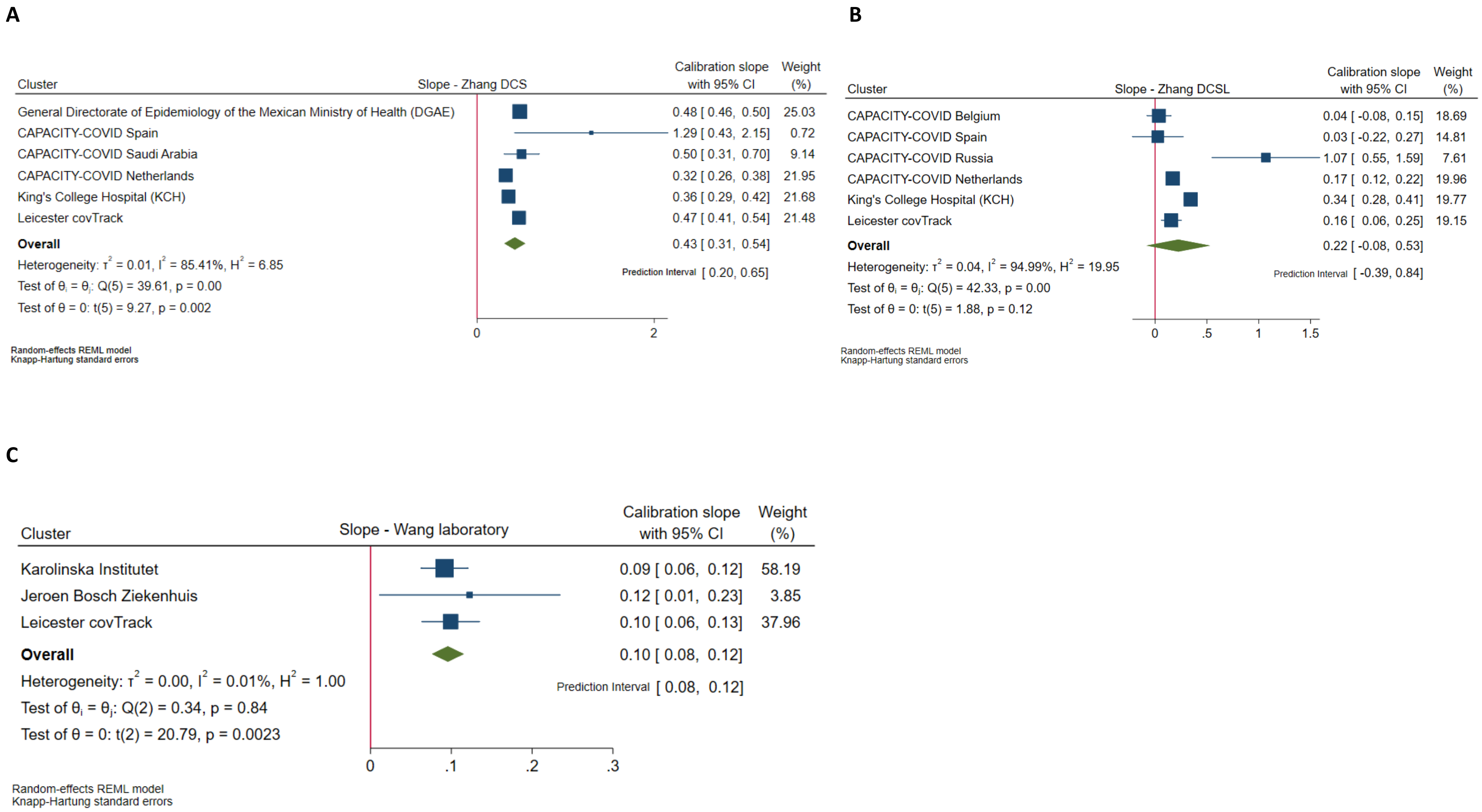

Figure S3: Pooled slope estimates with corresponding 95% CI and approximate 95% PI. A: Zhang DCS model. B: Zhang DCSL model. C: Wang laboratory model.

Calibration forest plots of the 4C Mortality Score, Wang clinical model, Xie model, and Hu model

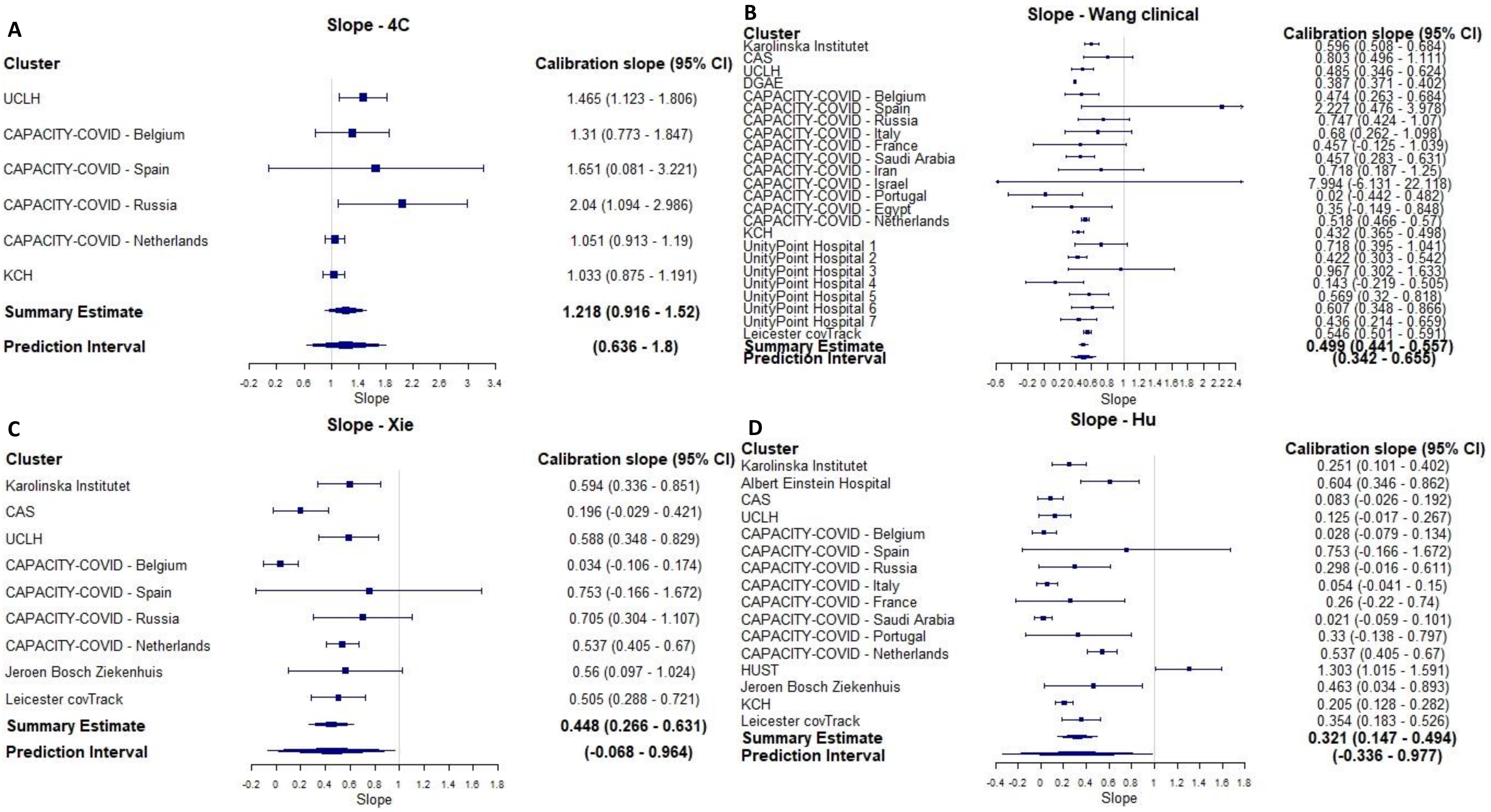

Figure S4: Pooled slope estimates with corresponding 95% CI and approximate 95% PI. A: 4C Mortality Score. B: Wang clinical model. C: Xie model. D: Hu model.

Calibration-in-the-large

Table S10: Meta-analysis results of the calibration-in-the-large.

Model	Estimate (95% CI) [95% PI]
Xie	-0.10 (-1.00 to 0.80) [-2.87 to 2.67]
Hu	-0.95 (-1.64 to -0.26) [-3.71 to 1.81]
Zhang DCS	1.57 (0.52 to 2.62) [-1.19 to 4.33]
Zhang DCSL	0.74 (0.37 to 1.12) [-0.18 to 1.67]
4C	-0.52 (-1.15 to 0.12) [-2.13 to 1.09]
Wang clinical	-0.72 (-1.10 to -0.33) [-2.56 to 1.13]
Wang laboratory	22.54 (17.52 to 27.56) [12.73 to 32.35]

Sensitivity analysis results

C-statistic point estimate

The point estimate of the discrimination was the highest for the 4C Mortality Score (reference), followed in order by the clinical model by Wang et al. (beta = -0.28, 95% CI -0.49 to -0.07), the model by Xie et al. (beta = -0.39, 95% CI -0.63 to -0.15), the DCS model by Zhang et al. (beta = -0.45, 95% CI -0.68 to -0.21), the model by Hu et al. (beta = -0.56, 95% CI -0.78 to -0.34), the score by Bello-Chavolla et al. (beta = -0.59, 95% CI -0.83 to -0.35), the DCSL model by Zhang et al. (beta = -0.75, 95% CI -0.99 to -0.50), and finally the laboratory model by Wang et al. (beta = -0.83, 95% CI -1.13 to -0.54). The point estimate for the discrimination was highest in Israel (beta = 2.69, 95% CI -0.38 to 5.77) and lowest in Mexico (beta = -0.32, 95% CI -0.52 to -0.12).

Table S11: Predicted c-statistics (95% CI) for each included model in each included country in the sensitivity analysis

Country	4C	Bello-Chavolla	Hu	Wang clinical	Wang laboratory	Xie	Zhang DCS	Zhang DCSL
Belgium	0.78(0.72 to 0.82)	0.66(0.59 to 0.72)	0.67(0.60 to 0.72)	0.73(0.67 to 0.78)	0.60(0.52 to 0.68)	0.70(0.64 to 0.76)	0.69(0.63 to 0.75)	0.62(0.55 to 0.69)
Brazil	0.90(0.77 to 0.96)	0.83(0.65 to 0.92)	0.83(0.67 to 0.92)	0.87(0.72 to 0.94)	0.79(0.59 to 0.91)	0.85(0.70 to 0.94)	0.85(0.69 to 0.93)	0.80(0.62 to 0.91)
China	0.97(0.95 to 0.98)	0.95(0.91 to 0.97)	0.95(0.92 to 0.97)	0.96(0.93 to 0.98)	0.94(0.89 to 0.96)	0.96(0.93 to 0.98)	0.96(0.92 to 0.97)	0.94(0.90 to 0.97)
Czech Republic	0.83(0.76 to 0.88)	0.73(0.64 to 0.80)	0.73(0.65 to 0.80)	0.78(0.71 to 0.84)	0.68(0.57 to 0.77)	0.76(0.69 to 0.83)	0.75(0.67 to 0.82)	0.69(0.60 to 0.78)
Egypt	0.74(0.48 to 0.90)	0.62(0.34 to 0.83)	0.62(0.35 to 0.83)	0.69(0.42 to 0.87)	0.56(0.29 to 0.80)	0.66(0.39 to 0.86)	0.65(0.38 to 0.85)	0.58(0.31 to 0.81)
France	0.84(0.65 to 0.93)	0.74(0.50 to 0.89)	0.74(0.51 to 0.89)	0.79(0.58 to 0.91)	0.69(0.44 to 0.86)	0.78(0.55 to 0.91)	0.77(0.54 to 0.90)	0.71(0.46 to 0.87)
Iran	0.80(0.68 to 0.89)	0.70(0.55 to 0.81)	0.70(0.55 to 0.82)	0.76(0.62 to 0.85)	0.64(0.48 to 0.78)	0.74(0.59 to 0.84)	0.73(0.58 to 0.83)	0.66(0.50 to 0.79)
Israel	0.98(0.73 to 1.00)	0.97(0.60 to 1.00)	0.97(0.61 to 1.00)	0.98(0.68 to 1.00)	0.96(0.54 to 1.00)	0.98(0.65 to 1.00)	0.97(0.64 to 1.00)	0.97(0.57 to 1.00)
Italy	0.76(0.65 to 0.85)	0.64(0.51 to 0.75)	0.65(0.53 to 0.75)	0.71(0.60 to 0.80)	0.58(0.44 to 0.71)	0.69(0.56 to 0.79)	0.68(0.55 to 0.78)	0.61(0.47 to 0.72)
Mexico	0.75(0.69 to 0.79)	0.62(0.57 to 0.67)	0.62(0.57 to 0.68)	0.69(0.64 to 0.73)	0.56(0.48 to 0.64)	0.66(0.60 to 0.72)	0.65(0.60 to 0.70)	0.58(0.52 to 0.64)
Netherlands	0.78(0.75 to 0.82)	0.67(0.62 to 0.71)	0.67(0.64 to 0.71)	0.73(0.70 to 0.76)	0.61(0.55 to 0.67)	0.71(0.67 to 0.75)	0.70(0.66 to 0.74)	0.63(0.59 to 0.68)
Portugal	0.68(0.50 to 0.81)	0.54(0.36 to 0.71)	0.55(0.37 to 0.71)	0.61(0.44 to 0.76)	0.48(0.30 to 0.66)	0.59(0.41 to 0.75)	0.58(0.40 to 0.74)	0.50(0.33 to 0.68)
Russia	0.91(0.87 to 0.93)	0.84(0.79 to 0.88)	0.84(0.79 to 0.89)	0.88(0.84 to 0.91)	0.81(0.73 to 0.86)	0.87(0.82 to 0.90)	0.86(0.81 to 0.90)	0.82(0.76 to 0.87)
Saudi Arabia	0.77(0.70 to 0.82)	0.65(0.57 to 0.72)	0.65(0.58 to 0.72)	0.71(0.65 to 0.77)	0.59(0.49 to 0.68)	0.69(0.62 to 0.76)	0.68(0.61 to 0.74)	0.61(0.53 to 0.69)
Spain	0.85(0.78 to 0.91)	0.76(0.66 to 0.84)	0.77(0.67 to 0.85)	0.82(0.73 to 0.88)	0.72(0.59 to 0.81)	0.80(0.70 to 0.87)	0.79(0.69 to 0.86)	0.73(0.63 to 0.82)
Sweden	0.86(0.83 to 0.88)	0.77(0.72 to 0.81)	0.77(0.73 to 0.81)	0.82(0.78 to 0.85)	0.72(0.66 to 0.77)	0.80(0.76 to 0.84)	0.79(0.75 to 0.83)	0.74(0.69 to 0.78)
United Kingdom	0.80(0.77 to 0.83)	0.69(0.65 to 0.73)	0.70(0.66 to 0.73)	0.75(0.73 to 0.78)	0.64(0.58 to 0.69)	0.73(0.69 to 0.77)	0.72(0.69 to 0.75)	0.66(0.61 to 0.70)
United States	0.84(0.80 to 0.88)	0.75(0.69 to 0.80)	0.75(0.70 to 0.80)	0.80(0.76 to 0.84)	0.70(0.62 to 0.77)	0.78(0.73 to 0.83)	0.78(0.72 to 0.82)	0.72(0.65 to 0.78)

Table S12: Predicted O:E ratios (95% CI) for each included model in each included country in the sensitivity analysis

**Country**	**4C**	**Bello-Chavolla**	**Hu**	**Wang clinical**	**Wang laboratory**	**Xie**	**Zhang DCS**	**Zhang DCSL**
Belgium	0.69(0.46 to 1.04)	0.13(0.05 to 0.33)	0.57(0.39 to 0.84)	0.54(0.37 to 0.79)	1322.58(302.22 to 5787.93)	0.95(0.64 to 1.41)	1.49(0.93 to 2.39)	1.47(0.96 to 2.25)
Brazil	0.23(0.09 to 0.57)	0.04(0.01 to 0.15)	0.19(0.08 to 0.44)	0.18(0.07 to 0.44)	437.89(81.7 to 2346.91)	0.31(0.13 to 0.78)	0.49(0.2 to 1.25)	0.49(0.19 to 1.23)
China	1.39(0.63 to 3.03)	0.26(0.08 to 0.8)	1.15(0.57 to 2.3)	1.09(0.51 to 2.31)	2665.33(533.29 to 13321.08)	1.91(0.89 to 4.13)	3.01(1.36 to 6.66)	2.97(1.34 to 6.56)
Czech Republic	0.86(0.5 to 1.46)	0.16(0.06 to 0.42)	0.71(0.44 to 1.13)	0.67(0.42 to 1.07)	1644.84(365.5 to 7402.2)	1.18(0.73 to 1.9)	1.85(1.07 to 3.22)	1.83(1.05 to 3.18)
Egypt	1.26(0.46 to 3.42)	0.24(0.07 to 0.84)	1.04(0.39 to 2.76)	0.99(0.39 to 2.5)	2418.41(431.1 to 13567.09)	1.74(0.65 to 4.67)	2.73(1 to 7.44)	2.69(0.98 to 7.38)
France	0.24(0.08 to 0.72)	0.04(0.01 to 0.17)	0.2(0.07 to 0.57)	0.19(0.06 to 0.54)	453.81(75.66 to 2721.79)	0.33(0.11 to 0.98)	0.51(0.17 to 1.56)	0.51(0.17 to 1.55)
Iran	0.52(0.2 to 1.34)	0.1(0.03 to 0.33)	0.43(0.17 to 1.08)	0.41(0.17 to 0.98)	1004.72(185.05 to 5455.14)	0.72(0.28 to 1.83)	1.13(0.44 to 2.92)	1.12(0.43 to 2.89)
Israel	0.71(0.1 to 5.03)	0.13(0.02 to 1.1)	0.59(0.08 to 4.12)	0.56(0.08 to 3.81)	1372.03(123.72 to 15216.16)	0.99(0.14 to 6.91)	1.55(0.22 to 10.93)	1.53(0.22 to 10.81)
Italy	0.76(0.4 to 1.46)	0.14(0.05 to 0.4)	0.63(0.35 to 1.13)	0.6(0.34 to 1.07)	1466.7(311.92 to 6896.79)	1.05(0.56 to 1.98)	1.65(0.85 to 3.2)	1.63(0.84 to 3.17)
Mexico	3.65(2 to 6.65)	0.69(0.35 to 1.36)	3.02(1.72 to 5.3)	2.86(1.7 to 4.81)	7016.94(1520.68 to 32378.57)	5.04(2.81 to 9.04)	7.91(4.7 to 13.31)	7.81(4.23 to 14.42)
Netherlands	0.84(0.58 to 1.21)	0.16(0.06 to 0.38)	0.69(0.51 to 0.94)	0.66(0.47 to 0.91)	1608.49(375.02 to 6899)	1.16(0.84 to 1.6)	1.81(1.23 to 2.67)	1.79(1.22 to 2.62)
Portugal	0.93(0.45 to 1.9)	0.17(0.06 to 0.51)	0.77(0.4 to 1.48)	0.73(0.38 to 1.4)	1787.76(369.11 to 8659.01)	1.28(0.64 to 2.59)	2.02(0.97 to 4.17)	1.99(0.96 to 4.12)
Russia	0.34(0.21 to 0.52)	0.06(0.02 to 0.16)	0.28(0.18 to 0.42)	0.26(0.17 to 0.4)	645.2(145.89 to 2853.49)	0.46(0.3 to 0.72)	0.73(0.44 to 1.21)	0.72(0.45 to 1.14)
Saudi Arabia	2.03(1.19 to 3.46)	0.38(0.15 to 0.98)	1.68(1.05 to 2.67)	1.59(1 to 2.52)	3898.48(865.71 to 17555.68)	2.8(1.67 to 4.7)	4.4(2.71 to 7.13)	4.34(2.5 to 7.53)
Spain	0.67(0.43 to 1.06)	0.13(0.05 to 0.32)	0.56(0.36 to 0.85)	0.53(0.34 to 0.81)	1293.26(292.08 to 5726.23)	0.93(0.6 to 1.45)	1.46(0.91 to 2.34)	1.44(0.89 to 2.32)
Sweden	0.65(0.43 to 0.99)	0.12(0.05 to 0.31)	0.54(0.36 to 0.8)	0.51(0.34 to 0.76)	1249.18(296.76 to 5258.28)	0.9(0.6 to 1.35)	1.41(0.86 to 2.3)	1.39(0.86 to 2.26)
United Kingdom	0.79(0.57 to 1.09)	0.15(0.06 to 0.36)	0.65(0.5 to 0.85)	0.62(0.47 to 0.81)	1518.77(359.18 to 6422.03)	1.09(0.81 to 1.48)	1.71(1.21 to 2.42)	1.69(1.2 to 2.38)
United States	0.67(0.42 to 1.09)	0.13(0.05 to 0.32)	0.56(0.36 to 0.86)	0.53(0.38 to 0.73)	1294.37(292.74 to 5723.16)	0.93(0.58 to 1.48)	1.46(0.89 to 2.39)	1.44(0.87 to 2.38)

Table S13: Predicted calibration slopes (95% CI) for each included model in each included country in the sensitivity analysis

**Country**	**4C**	**Bello-Chavolla**	**Hu**	**Wang clinical**	**Wang laboratory**	**Xie**	**Zhang DCS**	**Zhang DCSL**
Belgium	0.82(0.55 to 1.1)	0.31(-0.2 to 0.82)	0.07(-0.16 to 0.29)	0.37(0.14 to 0.6)	-0.05(-0.36 to 0.25)	0.27(0.03 to 0.51)	0.28(0 to 0.56)	0.01(-0.23 to 0.25)
Brazil	1.36(0.84 to 1.88)	0.85(0.18 to 1.52)	0.6(0.14 to 1.07)	0.91(0.41 to 1.4)	0.48(-0.05 to 1.02)	0.81(0.3 to 1.32)	0.82(0.3 to 1.33)	0.55(0.03 to 1.06)
China	2.06(1.52 to 2.6)	1.55(0.86 to 2.23)	1.3(0.82 to 1.79)	1.61(1.09 to 2.12)	1.18(0.63 to 1.73)	1.51(0.98 to 2.03)	1.52(0.98 to 2.05)	1.25(0.71 to 1.78)
Czech Republic	0.92(0.58 to 1.25)	0.4(-0.13 to 0.94)	0.16(-0.11 to 0.43)	0.46(0.19 to 0.74)	0.04(-0.31 to 0.39)	0.37(0.08 to 0.65)	0.38(0.05 to 0.7)	0.1(-0.22 to 0.43)
Egypt	0.8(0.1 to 1.5)	0.29(-0.52 to 1.09)	0.05(-0.63 to 0.72)	0.35(-0.31 to 1)	-0.08(-0.78 to 0.63)	0.25(-0.44 to 0.94)	0.26(-0.43 to 0.95)	-0.01(-0.7 to 0.68)
France	0.97(0.44 to 1.5)	0.46(-0.22 to 1.13)	0.21(-0.27 to 0.7)	0.52(0.03 to 1.01)	0.09(-0.45 to 0.63)	0.42(-0.1 to 0.94)	0.43(-0.09 to 0.95)	0.16(-0.37 to 0.68)
Iran	1.17(0.45 to 1.9)	0.66(-0.17 to 1.49)	0.41(-0.29 to 1.12)	0.72(0.03 to 1.4)	0.29(-0.44 to 1.03)	0.62(-0.1 to 1.34)	0.63(-0.09 to 1.35)	0.36(-0.36 to 1.08)
Israel	8.45(-6.87 to 23.76)	7.93(-7.38 to 23.25)	7.69(-7.62 to 23)	7.99(-7.32 to 23.3)	7.57(-7.74 to 22.88)	7.9(-7.42 to 23.21)	7.9(-7.41 to 23.22)	7.63(-7.68 to 22.95)
Italy	0.91(0.52 to 1.3)	0.39(-0.18 to 0.97)	0.15(-0.17 to 0.48)	0.46(0.11 to 0.8)	0.03(-0.38 to 0.44)	0.36(-0.02 to 0.73)	0.37(-0.02 to 0.75)	0.1(-0.29 to 0.48)
Mexico	0.93(0.58 to 1.28)	0.42(0.05 to 0.79)	0.17(-0.14 to 0.49)	0.48(0.2 to 0.76)	0.05(-0.31 to 0.42)	0.38(0.05 to 0.71)	0.39(0.11 to 0.67)	0.12(-0.22 to 0.46)
Netherlands	1.05(0.81 to 1.28)	0.53(0.05 to 1.02)	0.29(0.1 to 0.47)	0.59(0.41 to 0.78)	0.17(-0.08 to 0.42)	0.49(0.29 to 0.7)	0.5(0.28 to 0.72)	0.23(0.02 to 0.45)
Portugal	0.78(0.28 to 1.27)	0.26(-0.38 to 0.91)	0.02(-0.43 to 0.47)	0.32(-0.13 to 0.77)	-0.1(-0.61 to 0.41)	0.23(-0.25 to 0.71)	0.24(-0.25 to 0.72)	-0.04(-0.52 to 0.45)
Russia	1.33(0.99 to 1.66)	0.82(0.28 to 1.36)	0.57(0.28 to 0.86)	0.88(0.59 to 1.17)	0.45(0.09 to 0.81)	0.78(0.48 to 1.08)	0.79(0.46 to 1.12)	0.52(0.2 to 0.83)
Saudi Arabia	0.9(0.58 to 1.22)	0.39(-0.13 to 0.9)	0.14(-0.11 to 0.4)	0.45(0.19 to 0.71)	0.02(-0.32 to 0.36)	0.35(0.05 to 0.65)	0.36(0.08 to 0.63)	0.09(-0.22 to 0.4)
Spain	1.17(0.76 to 1.59)	0.66(0.07 to 1.25)	0.42(0.03 to 0.8)	0.72(0.33 to 1.11)	0.3(-0.14 to 0.73)	0.62(0.23 to 1.02)	0.63(0.23 to 1.03)	0.36(0 to 0.72)
Sweden	0.89(0.65 to 1.13)	0.38(-0.13 to 0.88)	0.13(-0.09 to 0.35)	0.44(0.22 to 0.65)	0.01(-0.24 to 0.27)	0.34(0.1 to 0.58)	0.35(0.08 to 0.62)	0.08(-0.19 to 0.34)
United Kingdom	1.01(0.8 to 1.23)	0.5(0.03 to 0.98)	0.26(0.1 to 0.41)	0.56(0.41 to 0.71)	0.14(-0.1 to 0.38)	0.46(0.27 to 0.65)	0.47(0.28 to 0.67)	0.2(0.01 to 0.4)
United States	0.96(0.66 to 1.26)	0.45(-0.05 to 0.95)	0.21(-0.05 to 0.46)	0.51(0.33 to 0.69)	0.09(-0.23 to 0.4)	0.41(0.13 to 0.69)	0.42(0.14 to 0.71)	0.15(-0.14 to 0.44)

Table S14: Predicted calibration-in-the-large (95% CI) for each included model in each included country in the sensitivity analysis

Country	4C	Bello-Chavolla	Hu	Wang clinical	Wang laboratory	Xie	Zhang DCS	Zhang DCSL
Belgium	-0.69(-1.84 to 0.46)	-1.89(-4.60 to 0.81)	-1.15(-2.23 to -0.07)	-1.35(-2.43 to -0.27)	22.41(20.77 to 24.06)	-0.16(-1.28 to 0.96)	0.52(-0.82 to 1.85)	0.73(-0.46 to 1.92)
Brazil	-2.37(-4.70 to -0.04)	-3.57(-6.91 to -0.22)	-2.82(-4.93 to -0.72)	-3.03(-5.29 to -0.78)	20.74(18.18 to 23.29)	-1.83(-4.13 to 0.47)	-1.16(-3.53 to 1.21)	-0.95(-3.31 to 1.42)
China	1.00(-1.28 to 3.28)	-0.20(-3.51 to 3.11)	0.54(-1.50 to 2.59)	0.34(-1.86 to 2.54)	24.10(21.59 to 26.61)	1.53(-0.71 to 3.78)	2.21(-0.11 to 4.52)	2.42(0.11 to 4.73)
Czech Republic	-0.39(-1.87 to 1.09)	-1.59(-4.39 to 1.21)	-0.85(-2.13 to 0.44)	-1.05(-2.34 to 0.23)	22.71(20.90 to 24.53)	0.14(-1.17 to 1.46)	0.82(-0.72 to 2.36)	1.03(-0.50 to 2.56)
Egypt	0.64(-1.77 to 3.05)	-0.56(-3.90 to 2.79)	0.19(-2.15 to 2.52)	-0.02(-2.21 to 2.17)	23.75(21.12 to 26.38)	1.18(-1.20 to 3.55)	1.85(-0.58 to 4.28)	2.06(-0.37 to 4.50)
France	-1.08(-2.92 to 0.76)	-2.28(-5.27 to 0.71)	-1.54(-3.18 to 0.11)	-1.74(-3.38 to -0.11)	22.02(19.91 to 24.14)	-0.55(-2.34 to 1.25)	0.13(-1.75 to 2.00)	0.34(-1.54 to 2.22)
Iran	-0.87(-3.22 to 1.48)	-2.07(-5.38 to 1.23)	-1.33(-3.60 to 0.95)	-1.53(-3.66 to 0.59)	22.23(19.66 to 24.81)	-0.34(-2.66 to 1.98)	0.34(-2.04 to 2.71)	0.55(-1.83 to 2.93)
Israel	-0.16(-2.93 to 2.60)	-1.36(-4.98 to 2.25)	-0.62(-3.32 to 2.08)	-0.83(-3.40 to 1.75)	22.94(19.98 to 25.90)	0.37(-2.37 to 3.11)	1.04(-1.74 to 3.83)	1.26(-1.54 to 4.05)
Italy	-0.68(-2.50 to 1.13)	-1.88(-4.86 to 1.09)	-1.14(-2.77 to 0.49)	-1.34(-2.95 to 0.26)	22.42(20.32 to 24.52)	-0.15(-1.92 to 1.63)	0.53(-1.33 to 2.38)	0.74(-1.12 to 2.60)
Mexico	2.40(0.67 to 4.14)	1.20(-0.82 to 3.22)	1.95(0.31 to 3.58)	1.74(0.22 to 3.26)	25.51(23.48 to 27.54)	2.94(1.24 to 4.63)	3.61(2.09 to 5.13)	3.82(2.05 to 5.60)
Netherlands	0.16(-0.86 to 1.19)	-1.04(-3.65 to 1.58)	-0.29(-1.16 to 0.58)	-0.50(-1.41 to 0.41)	23.27(21.85 to 24.69)	0.70(-0.22 to 1.62)	1.37(0.27 to 2.47)	1.59(0.51 to 2.67)
Portugal	0.01(-1.79 to 1.81)	-1.19(-4.16 to 1.77)	-0.45(-2.05 to 1.15)	-0.66(-2.25 to 0.94)	23.11(21.03 to 25.20)	0.54(-1.22 to 2.30)	1.21(-0.63 to 3.05)	1.43(-0.41 to 3.27)
Russia	-1.81(-3.00 to -0.63)	-3.01(-5.73 to -0.29)	-2.27(-3.40 to -1.13)	-2.47(-3.59 to -1.36)	21.29(19.62 to 22.96)	-1.28(-2.43 to -0.12)	-0.60(-1.98 to 0.77)	-0.39(-1.61 to 0.83)
Saudi Arabia	1.27(-0.23 to 2.77)	0.07(-2.68 to 2.83)	0.82(-0.48 to 2.12)	0.61(-0.68 to 1.90)	24.38(22.55 to 26.21)	1.81(0.36 to 3.26)	2.48(1.12 to 3.84)	2.70(1.15 to 4.24)
Spain	-0.47(-1.62 to 0.69)	-1.67(-4.34 to 1.01)	-0.92(-1.99 to 0.15)	-1.13(-2.20 to -0.06)	22.64(21.00 to 24.27)	0.07(-1.05 to 1.19)	0.74(-0.47 to 1.95)	0.96(-0.27 to 2.18)
Sweden	-1.37(-2.54 to -0.20)	-2.57(-5.28 to 0.14)	-1.82(-2.91 to -0.74)	-2.03(-3.12 to -0.94)	21.74(20.31 to 23.16)	-0.83(-1.96 to 0.29)	-0.16(-1.51 to 1.19)	0.05(-1.27 to 1.38)
United Kingdom	-0.66(-1.57 to 0.25)	-1.86(-4.43 to 0.71)	-1.12(-1.87 to -0.36)	-1.33(-2.08 to -0.57)	22.44(21.06 to 23.83)	-0.13(-0.98 to 0.73)	0.54(-0.44 to 1.53)	0.76(-0.21 to 1.73)
United States	-0.30(-1.59 to 0.99)	-1.50(-4.15 to 1.16)	-0.75(-1.89 to 0.38)	-0.96(-1.76 to -0.16)	22.81(21.14 to 24.47)	0.24(-0.99 to 1.47)	0.91(-0.42 to 2.24)	1.12(-0.22 to 2.47)

Supplementary Material E – Collaborators within the CAPACITY-COVID consortium

Al-Ali AK[1], Al-Muhanna FA[2], Al-Windy NYY[3], Almubarak YA[4], Alnafie AN[5], Alshahrani M[6], Alshehri AM[7], Anthonio RL[8], Asselbergs FW[9,10,11], Aujayeb A[12], ten Berg JM[13], van Boxem AJM[14], Captur G[11,15], Caputo M[16,17], Charlotte N[18], Dark P[19], De Sutter J[20,21], Delsing CE[22], Dorman HGR[23], Drost JT[24], Emans ME[25], Ferreira JB[26], Gabriel L[27], van Gilst WH[28], Groenemeijer BE[29], Haerkens-Arends HE[30], van der Harst P[9], Hedayat B[31], van der Heijden DJ[32], Hellou E[33], Hermanides RS[34], Hermans-van Ast JF[35], van Hessen MWJ[36], Heymans SRB[37,38,39], van der Horst ICC[40,41], van Ierssel SH[42], Jewbali LS[43,44], Kearney MT[45], van Kesteren HAM[46], Kietselaer BLJH[47], Koning AMH[48], Kopylov PY[49], Kuijper AFM[50], Kwakkel-van Erp JM[51], van der Linden MMJM[52], Linschoten M[9], Linssen GCM[53], Macias Ruiz R[54], Magdelijns FJH[55], Martens FMAC[56], McCann GP[57], van der Meer P[58], Meijs MFL[59], Messiaen P[60,61], Monraats PS[62], Montagna L[63], Moriarty A[64], Mosterd A[65], Nierop PR[66], van Ofwegen-Hanekamp CEE[67], Pinto YM[68], Poorhosseini H[69], Prasad S[70,71], Redón J[72,73], Reidinga AC[74], Ribeiro MIA[75], Ripley DP[76], Salah R[77], Saneei E[78], Saxena M[79], Schaap J[80,81], Schellings DAAM[82], Schut A[80], Shafiee A[83], Shore AC[84], Siebelink HJ[85], van Smeden M[86], Smits PC[87], Pisters R[88], Tessitore E[89], Tieleman RG[28,90], Timmermans P Jr[91], Tio RA[92,93], Tjong FVY[68,94,95], den Uil CA[43,44,96], Van Craenenbroeck EM[97], van Veen HPAA[98], Veneman T[99], Verschure DO[100], de Vries JK[101], van de Wal RMA[102], van de Watering DJ[103], Westendorp ICD[104], Westendorp PHM[105], Weytjens C[106], Wierda E[95], Williams B[107], Woudstra P[108], Wu KW[109], Zaal R[110], Zaman AG[111], van der Zee PM[112] (listed alphabetically)

1. Department of Clinical Biochemistry, King Fahd Hospital of the University, Imam Abdulrahman Bin Faisal University, Alkhobar, Saudi Arabia
2. Department of Internal Medicine, King Fahd Hospital of the University, Imam Abdulrahman Bin Faisal University, Alkhobar, Saudi Arabia
3. Department of Cardiology, Gelre Hospital Zutphen, Zutphen, the Netherlands
4. Department of Critical Care, King Fahd Hospital of the University, Imam Abdulrahman Bin Faisal University, Alkhobar, Saudi Arabia
5. Department of Pathology, King Fahd Hospital of the University, Imam Abdulrahman Bin Faisal University, Alkhobar, Saudi Arabia
6. Department of Emergency Medicine, King Fahd Hospital of the University, Imam Abdulrahman Bin Faisal University, Alkhobar, Saudi Arabia
7. Department of Internal Medicine, Cardiology Section, King Fahd Hospital of the University, Imam Abdulrahman Bin Faisal University, Alkhobar, Saudi Arabia
8. Department of Cardiology, Treant Zorggroep, Emmen, the Netherlands

9. Department of Cardiology, Division of Heart and Lungs, University Medical Center Utrecht, Utrecht University, Utrecht, the Netherlands
10. Health Data Research United Kingdom and Institute of Health Informatics, University College London, London, United Kingdom
11. Institute of Cardiovascular Science, Faculty of Population Health Sciences, University College London, London, United Kingdom
12. Department of Respiratory and Acute Medicine, Northumbria Healthcare NHS Foundation Trust, Newcastle, United Kingdom
13. Department of Cardiology, St. Antonius Hospital, Nieuwegein, the Netherlands
14. Department of Pulmonology, Bravis Hospital, Roosendaal, the Netherlands
15. Department of Cardiology, Royal Free London NHS Foundation Trust, London, United Kingdom
16. Bristol Heart Institute, University Hospitals Bristol and Weston NHS Foundation Trust, Bristol, United Kingdom
17. Bristol Medical School, University of Bristol, Bristol, United Kingdom
18. Department of Cardiology, SSR Val Rosay, Saint Didier au Mont d'Or, Franc
19. Department of Critical Care, Salford Royal NHS Foundation Trust, Salford, United Kingdom
20. Department of Cardiology, AZ Maria Middelares, Ghent, Belgium
21. Department of Internal Medicine, Ghent University, Ghent, Belgium
22. Department of Internal Medicine and Infectious Diseases, Medisch Spectrum Twente, Enschede, the Netherlands
23. Department of Cardiology, Bravis Hospital, Roosendaal, the Netherlands
24. Department of Cardiology, Saxenburgh Medical Center, Hardenberg, the Netherlands
25. Department of Cardiology, Ikazia Hospital, Rotterdam, the Netherlands
26. Department of Cardiology, Hospital Professor Doutor Fernando Fonseca, Amadora, Portugal
27. Department of Cardiology, CHU UCL Namur site Godinne, Université Catholique de Louvain, Yvoir, Belgium
28. Department of Cardiology, University Medical Center Groningen, Groningen, the Netherlands
29. Department of Cardiology, Gelre Hospital Apeldoorn, Apeldoorn, the Netherlands
30. Department of Cardiology, Jeroen Bosch Hospital, 's-Hertogenbosch, the Netherlands
31. Department of Cardiology, Tehran Heart Center, Cardiovascular Diseases Research Institute, Tehran University of Medical Sciences, Tehran, Iran
32. Department of Cardiology, Haaglanden Medical Center, the Hague, the Netherlands

33. Department of Cardiology, E.M.M.S Hospital, Nazareth, Israel
34. Department of Cardiology, Isala Hospital, Zwolle, the Netherlands
35. Durrer Center, Netherlands Heart Institute, Utrecht, the Netherlands
36. Department of Cardiology, Groene Hart Hospital, Gouda, the Netherlands
37. Department of Cardiology, Cardiovascular Research Institute Maastricht (CARIM), Maastricht University Medical Center+, Maastricht, the Netherlands
38. Department of Cardiovascular Sciences, Center for Molecular and Vascular Biology, KU Leuven, Belgium
39. The Netherlands Heart Institute, Utrecht, the Netherlands
40. Department of Intensive Care, Maastricht University Medical Center+, Maastricht University, Maastricht, the Netherlands
41. Cardiovascular Research Institute Maastricht (CARIM), Maastricht University Medical Center, Maastricht, the Netherlands
42. Department of General Internal Medicine, Infectious Diseases and Tropical Medicine, Antwerp University Hospital, Antwerp, Belgium
43. Department of Cardiology, Erasmus MC University Medical Center, Rotterdam, the Netherlands
44. Department of Intensive Care, Erasmus MC University Medical Center, Rotterdam, the Netherlands
45. Leeds Institute for Cardiovascular and Metabolic Medicine, University of Leeds, Leeds, United Kingdom
46. Department of Cardiology, Admiraal de Ruyter Hospital, Goes, the Netherlands
47. Department of Cardiology, Zuyderland Medical Center, Heerlen, the Netherlands
48. Department of Gynaecology, Amstelland Hospital, Amstelveen, the Netherlands
49. World-Class Research Center Digital Biodesign and Personalized Healthcare, I.M. Sechenov First Moscow State Medical University, Sechenov University, Moscow, Russia
50. Department of Cardiology, Spaarne Gasthuis, Haarlem, the Netherlands
51. Department of Pulmonology, Antwerp University Hospital, University of Antwerp, Edegem, Belgium
52. Department of Cardiology, Franciscus Vlietland, Schiedam, the Netherlands
53. Department of Cardiology, Ziekenhuis Groep Twente (ZGT), Almelo, the Netherlands
54. Arrhythmias Unit, Department of Cardiology, Hospital Universitario Virgen de las Nieves, Granada, Spain
55. Department of Internal Medicine, Division of General Internal Medicine, Section Geriatric Medicine, Cardiovascular Research Institute Maastricht (CARIM), Maastricht University Medical Center+, Maastricht, the Netherlands

56. Department of Cardiology, Deventer Hospital, Deventer, the Netherlands
57. Department of Cardiovascular Sciences, University of Leicester and Cardiovascular Theme, National Institute for Health Research (NIHR) Leicester Biomedical Research Center, Glenfield Hospital, Leicester, United Kingdom
58. Department of Cardiology, LangeLand Hospital, Zoetermeer, the Netherlands
59. Department of Cardiology, Thorax Center Twente, Medisch Spectrum Twente, Enschede, the Netherlands
60. Department of Infectious Diseases & Immunity, Jessa Hospital, Hasselt, Belgium
61. Faculty of Medicine and Life Sciences, Hasselt University, Hasselt, Belgium
62. Department of Cardiology, Elizabeth-TweeSteden Hospital, Tilburg, the Netherlands
63. Department of Cardiology, A.O.U. San Luigi Gonzaga, Orbassano, Turin, Italy
64. Cardiovascular Research Unit, Craigavon Area Hospital, Southern Health and Social Care Trust, Portadown, Nothern Ireland
65. Department of Cardiology, Meander Medical Center, Amersfoort, the Netherlands
66. Department of Cardiology, Franciscus Gasthuis, Rotterdam, the Netherlands
67. Department of Cardiology, Diakonessenhuis, Utrecht, the Netherlands
68. Amsterdam University Medical Center, University of Amsterdam, Heart Center; Department of Clinical and Experimental Cardiology, Amsterdam Cardiovascular Sciences, Amsterdam, the Netherlands
69. Department of Interventional Cardiology, Tehran Heart Center, Cardiovascular Diseases Research Institute, Tehran University of Medical Sciences, Tehran, Iran
70. National Heart and Lung Institute, Imperial College, London, United Kingdom
71. Royal Brompton Hospital, London, United Kingdom
72. Department of Internal Medicine, Clinic University Hospital, INCLIVA Health Research Institute, Valencia, Spain
73. Department of Medicine, School of Medicine, University of Valencia, Valencia, Spain
74. Department of Intensive Care, Martini Hospital, Groningen, the Netherlands
75. Intensive Care Unit, Hospital do Espírito Santo, Évora, Portugal
76. Department of Cardiology, Northumbria Healthcare NHS Foundation Trust, Newcastle, United Kingdom
77. Benha Faculty of Medicine, Benha, Egypt

78. Department of Nursing, Tehran Heart Center, Cardiovascular Diseases Research Institute, Tehran University of Medical Sciences, Tehran, Iran
79. Barts National Institute for Health Research (NIHR) Biomedical Research Center, William Harvey Research Institute, Queen Mary University of London, United Kingdom
80. The Dutch Network for Cardiovascular Research (WCN), Utrecht, the Netherlands
81. Department of Cardiology, Amphia Hospital, the Netherlands
82. Department of Cardiology, Slingeland Hospital Doetinchem, the Netherlands
83. Department of Cardiovascular Research, Tehran Heart Center, Cardiovascular Diseases Research Institute, Tehran University of Medical Sciences, Tehran, Iran
84. National Institute for Health Research (NIHR) Exeter Clinical Research Facility, Royal Devon and Exeter Hospital and University of Exeter College of Medicine & Health, Exeter, United Kingdom
85. Department of Cardiology, Leiden University Medical Center, Leiden, the Netherlands
86. Julius Center for Health Sciences and Primary Care, University Medical Center Utrecht, Utrecht University, Utrecht, the Netherlands
87. Department of Cardiology, Maasstad Hospital, Rotterdam, the Netherlands
88. Department of Cardiology, Rijnstate Hospital, Arnhem, the Netherlands
89. Department of Cardiology, University Hospitals of Geneva, Geneva, Switzerland
90. Department of Cardiology, Martini Hospital, Groningen, the Netherlands
91. Department of Cardiology, Heart Center Hasselt, Jessa Hospital, Hasselt, Belgium
92. Department of Cardiology, Catharina Hospital, Eindhoven, the Netherlands
93. Department of Educational Development and Research in the Faculty of Health, Medicine and Life Sciences, Catharina Hospital, Eindhoven, the Netherlands
94. Department of Cardiology, Vrije Universiteit Amsterdam, Amsterdam Cardiovascular Sciences, Amsterdam, the Netherlands
95. Department of Cardiology, Dijklander Hospital, Hoorn, the Netherlands
96. Department of Intensive Care Medicine, Maasstad Hospital, Rotterdam, the Netherlands
97. Cardiovascular Research, Antwerp University and Cardiology, Antwerp University Hospital, Antwerp, Belgium
98. Department of Pulmonology, Medisch Spectrum Twente, Enschede, the Netherlands

99. Department of Intensive Care, Ziekenhuis Groep Twente (ZGT), Almelo, the Netherlands
100. Department of Cardiology, Zaans Medical Center, Zaandam, the Netherlands
101. Department of Internal Medicine, Antonius Hospital, Sneek, the Netherlands
102. Department of Cardiology, Bernhoven Hospital, Uden, the Netherlands
103. Department of Cardiology, Albert Schweitzer Hospital, Dordrecht, the Netherlands
104. Department of Cardiology, Rode Kruis Hospital, Beverwijk, the Netherlands
105. Department of Cardiology, Beatrix Hospital, Gorinchem, the Netherlands
106. Department of Cardiology, CHVZ, University Hospital Brussels, Jette, Belgium
107. National Institute for Health Research Biomedical Research Center, University College London Hospitals, London, United Kingdom
108. Department of Cardiology, Medical Center Leeuwarden (MCL), Leeuwarden, the Netherlands
109. Department of Cardiology, van Weel-Bethesda Hospital, Dirksland, the Netherlands
110. Department of Pulmonology, Ziekenhuis Groep Twente (ZGT), Almelo, the Netherlands
111. Freeman Hospital, Newcastle Upon Tyne NHS Hospitals Foundation Trust and Newcastle University, Newcastle Upon Tyne, NE7 7DN, United Kingdom
112. Department of Cardiology, St. Jansdal Hospital, Harderwijk, the Netherlands

Supplementary Material F – CovidRetro collaboration

Kristina Christozova (a), Karel Fiser (b), Jan Horejsek (c), Kristian Hrusak (a), Tomas Hyanek (d), Adam Jaros (d), Martin Jedlicka (e), Miroslav Kubiska (f), Viktor Kubricht (g), Luis F. Casas-Mendez (h), Grigorij Meseznikov (i), Adriana Mifkova (e), Martin Modrák (j), Rebeka Pokrievkova (k), Zbysek Pospisil (l), Robin Sin (f), Tomas Slisz (m), Daniel Suk (c), Jan Taborsky (n), Jana Vachalova (f), Jaromir Vajter (i), Martina Vasakova (m), Marketa Veverkova (o), Julie Vohryzkova (a).

a. 2nd Faculty of Medicine, Charles University in Prague
b. Department of Bioinformatics, 2nd Faculty of Medicine, Charles University in Prague
c. General University Hospital in Prague, Czech Republic
d. Na Homolce Hospital, Prague, Czech Republic
e. Military Hospital Olomouc, Olomouc, Czech Republic
f. Department of Infectious Diseases and Travel Medicine, Faculty of Medicine in Pilsen, Charles University, University Hospital in Pilsen, Pilsen, Czech Republic
g. Kralovske Vinohrady University Hospital, Prague, Czech Republic
h. Pneumology Department, Motol University Hospital, 2nd Faculty of Medicine Charles University in Prague. Prague, Czech Republic
i. Motol University Hospital, Prague, Czech Republic
j. Institute of Microbiology of the Czech Academy of Sciences, Prague, Czech Republic
k. 3rd Faculty of Medicine, Charles University in Prague
l. Trebic Hospital, Trebic, Czech Republic
m. Department of Respiratory Medicine, 1st Faculty of Medicine, Charles University; Thomayer Hospital, Prague, Czech Republic
n. AGEL Hospital Novy Jicin, Novy Jicin, Czech Republic
o. Horovice Hospital, Horovice, Czech Republic

Supplementary Material G – Comparison between development and validation cohorts

Table S15: Overview of sample characteristics for development and validation cohorts.

Cluster	Country	Participant numberSample size	Age – Mean [IQR]	Male sex	Recruitment dates
Wang clinical and laboratory model	China	D: 296<br>V: 44	D: 47.32 [32.37 to 62.27]*<br>V: 55.20 [38.4 to 72]*]	D: 140 (47.30%)<br>V: 156 (52.70%)	D: 7 Jan 2020 to 11 Feb 2020<br>V: 1 Jan 2020 to 20 Feb 2020
Bello-Chavolla*	Mexico	D: 41 307<br>V: 10 326	46.65 [30.82 to 62.48]*	29 803 (57.72%)	Before 18 May 2020
Xie model	China	D: 299<br>V: 145	D: 65 [54 to 73]*<br>V: 56 [47 to 68]*	D: 48.2%<br>V: 65.5%	D: Jan and Feb 2020<br>V: Before 28 Mar 2020
Hu model	China	D: 183<br>V: 64	D: Between 60.54 [47.35 to 73.73]* and 68.44 [58.5 to 78.38]*<br>V: Unknown	D: 107<br>V: Unknown	28 Jan 2020 to 11 Mar 2020
Zhang models	D: China<br>V: United Kingdom	D: 775<br>V: 226	D: 61 [50 to 68]*<br>V: 74 [59 to 85]*	D: 48.9%<br>V: 54.9%	Before 28 Apr 2020
4C Mortality Score	United Kingdom	D: 35 463<br>V: 22 361	D: 73 [59 to 83]*<br>V: 76 [60 to 85]*	D: 20 722 (58.43%)<br>V: 12 183 (54.48%)	D: 6 Feb 2020 to 20 May 2020<br>V: 21 May 2020 to 29 Jun 2020
Karolinska Institutet	Sweden	1670	57.30 [43 to 71]	983 (58.90%)	27 Feb 2020 to 1 Sep 2020
Czech Republic Academy of Sciences	Czech Republic	213	68.56 [58 to 80]	105 (49%)	3 Mar 2020 to 12 Oct 2020

UCLH	United Kingdom	411	66 [53 to 79]*	252 (61.31%)	1 Feb 2020 to 30 Apr 2020
DGAE	Mexico	28176	58.57 [48 to 70]	17019 (60.40%)	1 Mar 2020 to 16 Apr 2020
CAPACITY-COVID	Belgium	221	68.14 [57 to 81]	137 (61.99%)	12 Feb 2020 to 14 Oct 2020
	Spain	47	70.98 [55 to 83.75]	28 (59.57%)	5 Mar 2020 to 20 Apr 2020
	Russia	278	60.09 [50.25 to 71]	137 (49.28%)	22 Apr 2020 to 4 Jun 2020
	Italy	106	70.72 [62.25 to 78.75]	72 (67.92%)	6 Feb 2020 to 4 May 2020
	France	46	67.96 [62 to 77]	34 (73.91%)	13 Feb 2020 to 18 Dec 2020
	Saudi Arabia	389	50.56 [38 to 62]	270 (69.41%)	29 Feb 2020 to 24 Sep 2020
	Iran	90	63.19 [53.25 – 73]	59 (65.56%)	10 Feb 2020 to 5 May 2020
	Israel	25	50.36 [31 to 57]	14 (56%)	10 Apr 2020 to 9 Aug 2020
	Portugal	44	71.43 [63.75 to 82]	30 (61.18%)	25 Mar 2020 to 19 Aug 2020
	Egypt	45	60.89 [50 to 73]	21 (46.67%)	12 Apr 2020 to 12 Aug 2020

	Netherlands	5100	66.37 [57 to 76]	3172 (62.20%)	22 Nov 2019 to 30 Jul 2020
King’s College London	United Kingdom	2400	59.76 [47 to 76]	1314 (54.75%)	28 Feb 2020 to 28 Mar 2021
Albert Einstein Hospital	Brazil	453	56.44 [46 to 68.50]	105 (49%)	27 Feb 2020 to 25 Jun 2020
Huazhong University of Science and Technology (HUST)	China	332	58.98 [46 to 70]	198 (59.64%)	10 Jan 2020 to 18 Feb 2020
Jeroen Bosch Ziekenhuis	Netherlands	383	70.21 [61 to 81]	226 (59.01%)	9 Mar 2020 to 29 Dec 2020
Leicester covTrack	United Kingdom	3908	63.29 [50 to 79]	2063 (52.79%)	Jan 2020 to Apr 2021
UnityPoint Hospitals	United States (Iowa)	288	46.49 [32 to 59]	147 (51.04%)	3 Mar 2020 to 31 Jul 2020
		929	50.12 [33 to 68]	454 (48.87%)	
		95	45.31 [27 to 59]	45 (47.37%)	
		66	48.77 [31 to 65]	39 (59.09%)	
		511	51.03 [35 to 67]	240 (46.97%)	
		393	45.35 [30 to 60]	176 (44.78%)	
		295	47.60 [32 to 63]	162 (54.92%)	

Sample size: D = Development data, V = Validation data

* Age given as Median [IQR]

Supplement H – Summary of fitted parameters

Table S16: Fitted coefficients and standard error of each performance measure per model.

	C-statistic (SE)	Calibration slope (SE)	O:E ratio (SE)	Calibration-in-the-large (SE)
Bello-Chavolla	0.74 (0.09)			
Xie	1.08 (0.13)	0.45 (0.08)	-0.05 (0.21)	-0.10 (0.39)
Hu	1.04 (0.17)	0.32 (0.08)	-0.50 (0.17)	-0.95 (0.32)
Zhang DCS	0.84 (0.10)	0.43 (0.05)	0.95 (0.27)	1.57 (0.41)
Zhang DCSL	0.69 (0.22)	0.22 (0.12)	0.40 (0.07)	0.74 (0.15)
4C Mortality	1.37 (0.10)	1.22 (0.12)	-0.35 (0.18)	-0.52 (0.25)
Wang clinical	1.20 (0.09)	0.50 (0.03)	-0.43 (0.11)	-0.72 (0.18)
Wang laboratory	0.70 (0.06)	0.10 (0.00)	6.94 (1.09)	22.54 (1.17)

Supplement I

Table S17: Availability of model predictors per cluster

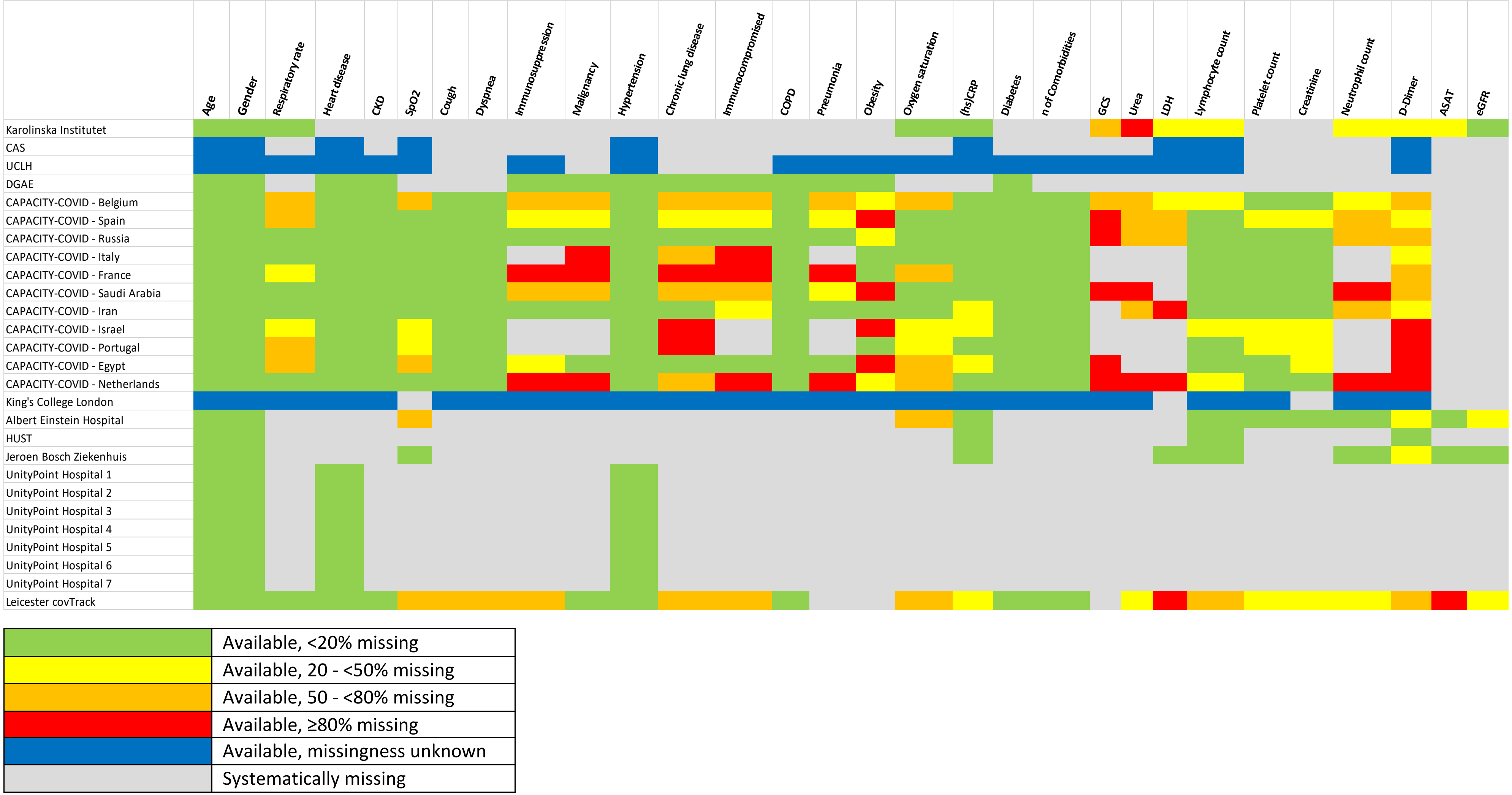

Supplement J – Calibration plots of the 4C Mortality Score

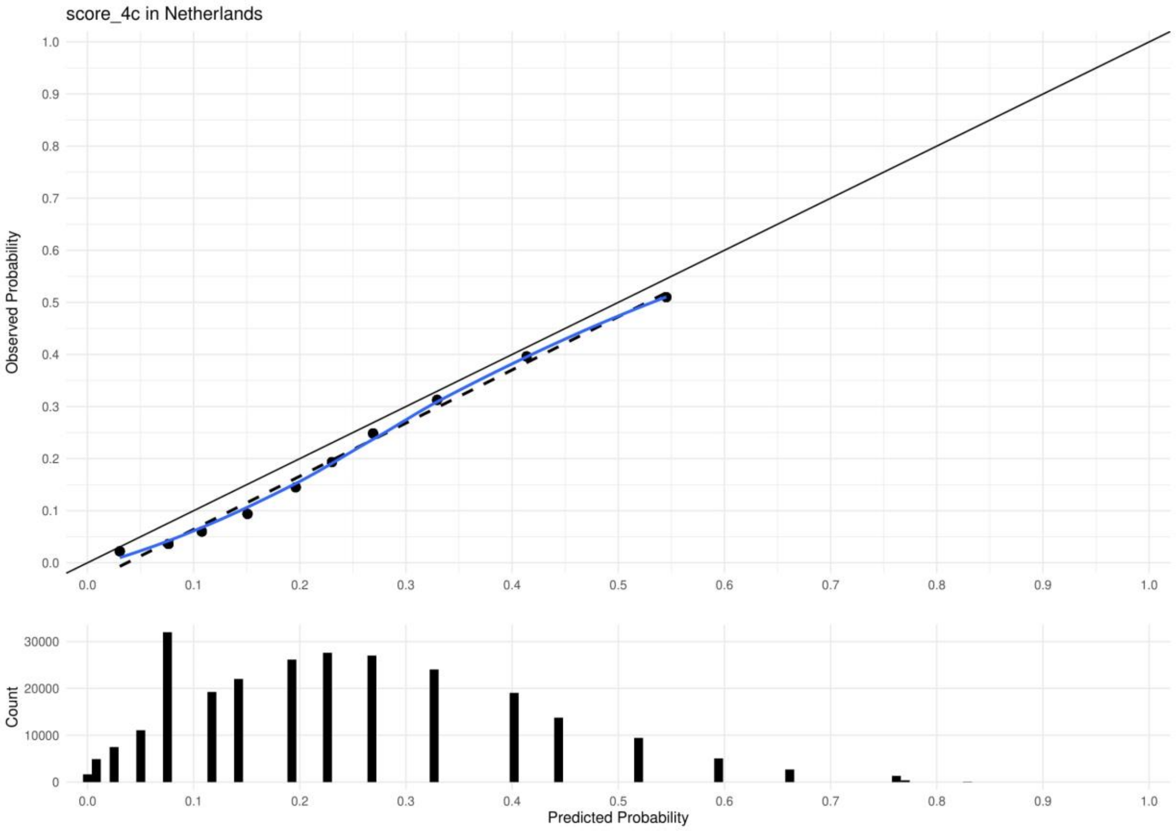

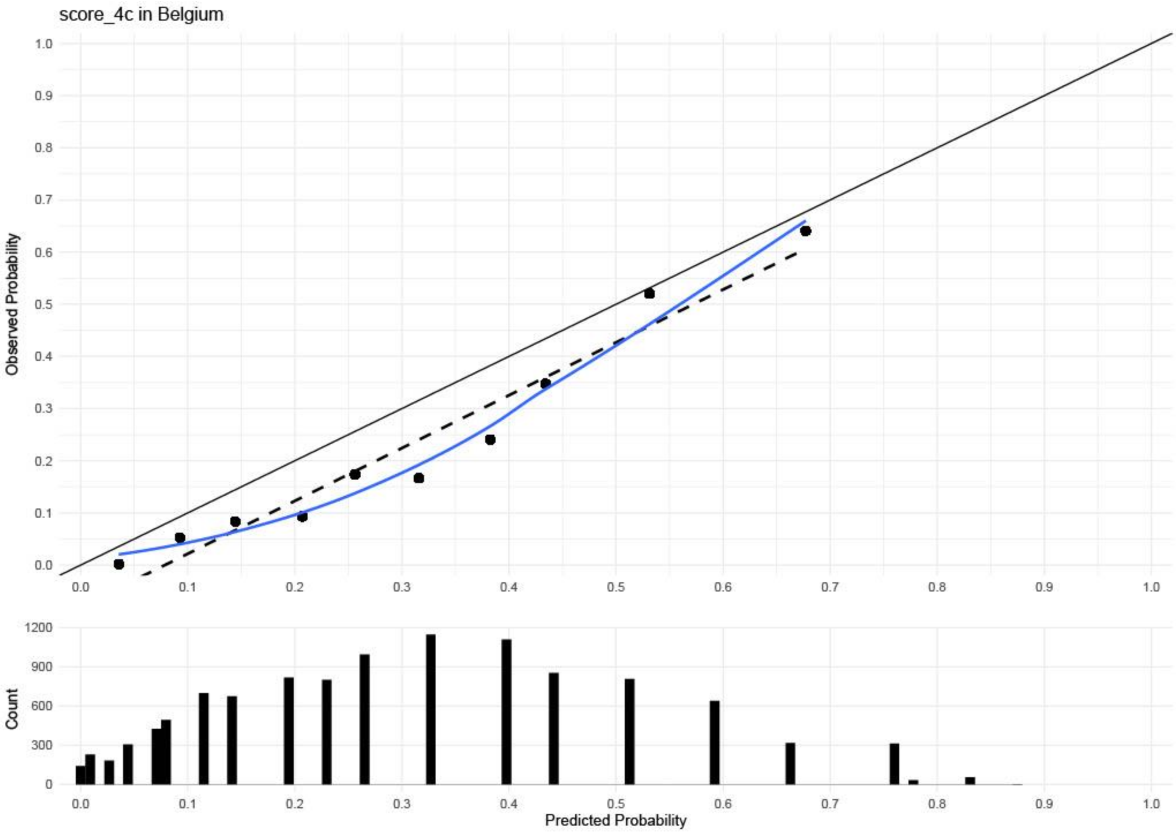

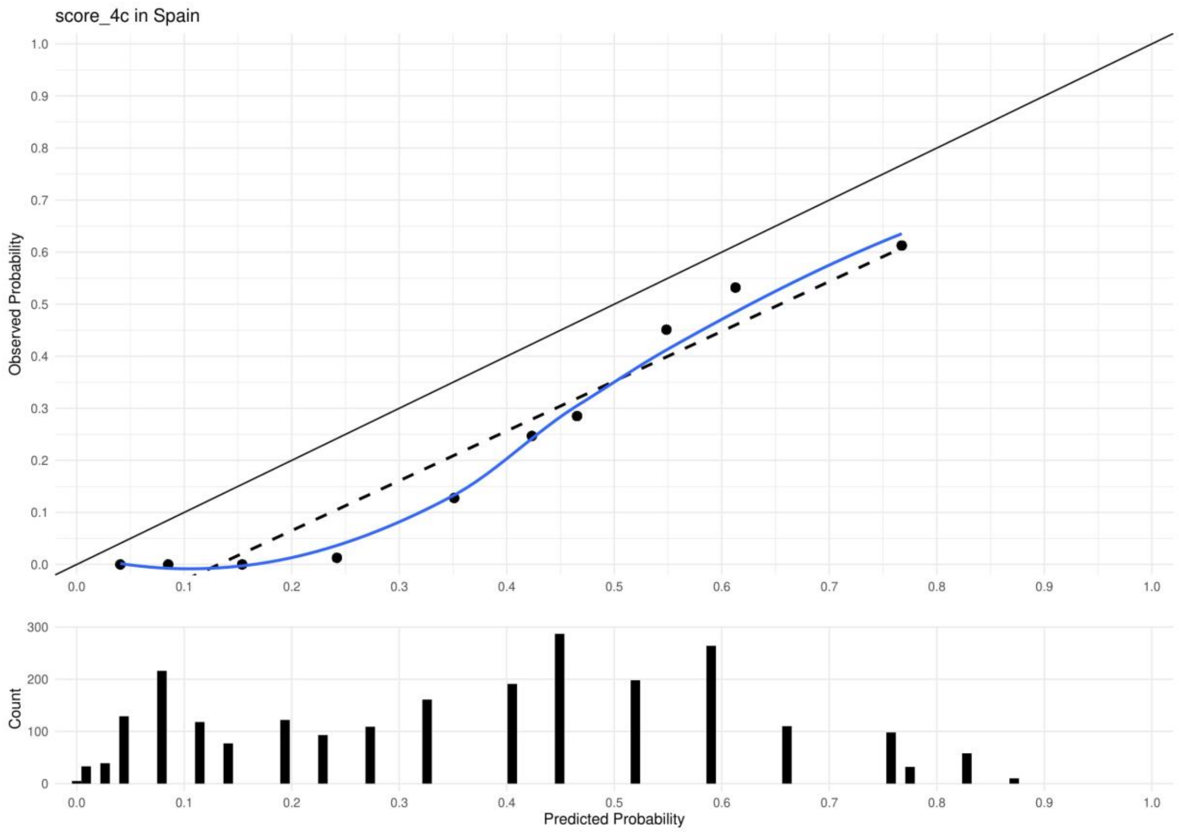
score_4c in Spain
Observed Probability
Count
Predicted Probability

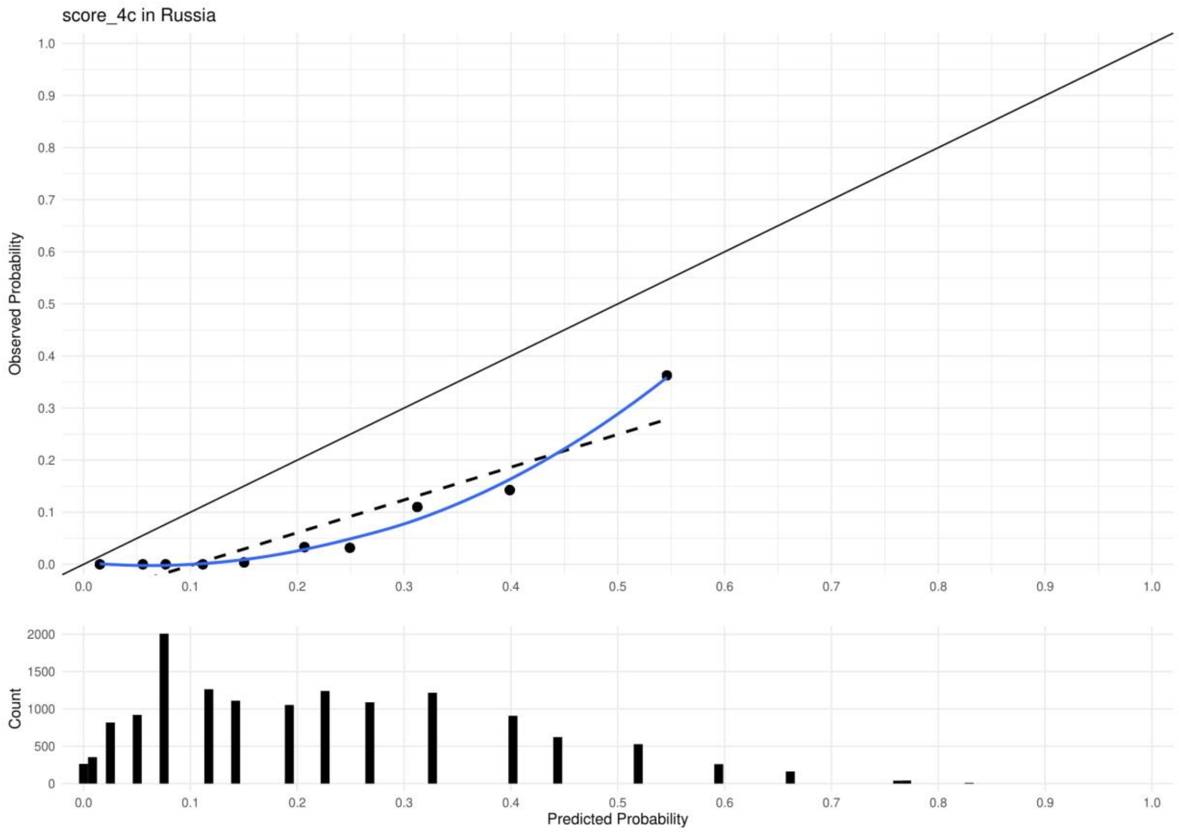
score_4c in Russia
Observed Probability
Count
Predicted Probability
